# Comparative Effectiveness of Nutritional Supplements in the Treatment of Knee Osteoarthritis: A Network Meta-Analysis

**DOI:** 10.3390/nu17152547

**Published:** 2025-08-03

**Authors:** Yuntong Zhang, Yunfei Gui, Roger Adams, Joshua Farragher, Catherine Itsiopoulos, Keegan Bow, Ming Cai, Jia Han

**Affiliations:** 1 Graduate School of Shanghai University of Traditional Chinese Medicine, Shanghai 201203, China; zhangyuntongcyr@163.com; 2 College of Rehabilitation Sciences, Shanghai University of Medicine and Health Sciences, Shanghai 201318, China; 243352500@st.usst.edu.cn (Y.G.); roger.adams@canberra.edu.au (R.A.); caim_16@sumhs.edu.cn (M.C.); 3 Research Institute for Sport and Exercise, University of Canberra, Canberra 2617, Australia; 4 School of Health and Biomedical Sciences, RMIT University, Melbourne 3000, Australia; joshua.farragher@rmit.edu.au (J.F.); catherine.itsiopoulos@rmit.edu.au (C.I.); keegan.bow@rmit.edu.au (K.B.)

**Keywords:** knee osteoarthritis, nutritional supplements, network meta-analysis

## Abstract

**Background**: Knee osteoarthritis (KOA) is a prevalent degenerative joint disease that can greatly affect quality of life in middle-aged and elderly individuals. Nutritional supplements are increasingly used for KOA due to their low risk, but direct comparative evidence on their efficacy and safety remains scarce. This study aimed to systematically compare the effectiveness and safety of seven common nutritional supplements for KOA. **Methods**: A systematic review and network meta-analysis were conducted following PRISMA guidelines. Embase, PubMed, and the Cochrane Library were searched through December 2024 for randomized controlled trials (RCTs) evaluating use of eggshell membrane, vitamin D, Boswellia, curcumin, ginger, krill oil, or collagen, versus placebo, in adults with KOA. Primary outcomes included changes in scores for WOMAC pain, stiffness and function, and pain visual analog scale (VAS). Adverse events were also assessed. Bayesian network meta-analyses estimated ranking probabilities for each intervention. **Results**: In total, 39 RCTs (42 studies; 4599 patients) were included. Compared with placebo, Boswellia showed significant improvements in WOMAC pain (mean difference [MD] = 10.58, 95% CI: 6.45 to 14.78, *p* < 0.05), stiffness (MD = 9.47, 95% CI: 6.39 254 to 12.74, *p* < 0.05), function (MD = 14.00, 95% CI: 7.74 to 20.21, *p* < 0.05), and VAS pain (MD = 17.26, 95% CI: 8.06 to 26.52, *p* < 0.05). Curcumin, collagen, ginger, and krill oil also demonstrated benefits in some outcomes. No supplement was associated with increased adverse events compared to placebo. Bayesian rankings indicated Boswellia had the highest probability of being most effective for pain and stiffness, with krill oil and curcumin showing potential for function improvement. **Conclusions**: Nutritional supplements, particularly Boswellia, appear to be effective and well-tolerated for improving KOA symptoms and function. These results suggest that certain supplements may be useful as part of non-pharmacological KOA management. However, further large-scale, well-designed randomized controlled trials (RCTs) are needed to confirm these findings, particularly those that include more standardized dosages and formulations, as well as to evaluate their long-term efficacy.

## 1. Introduction

Knee osteoarthritis (KOA) is a degenerative joint disease characterized by cartilage degradation and synovial inflammation, which typically presents with joint stiffness, swelling, pain, and restricted mobility [1]. With increasing life expectancy worldwide, KOA has emerged as the fourth leading cause of disability, posing a significant threat to the quality of life among older adults and placing a considerable burden on public health systems [2]. Current clinical guidelines advocate the use of acetaminophen and nonsteroidal anti-inflammatory drugs (NSAIDs) as first-line therapies for KOA [3,4]. However, prolonged NSAID use is associated with various adverse effects, including gastrointestinal complications, cardiovascular disease, and potential renal and hepatic toxicity [5,6]. As a result, many patients with KOA seek alternative or non-pharmacological therapies to relieve pain and functional impairment [7,8].

In recent years, there has been growing interest in the role of dietary and nutritional interventions for KOA, owing to their anti-inflammatory properties and potential therapeutic benefits [9,10]. An increasing body of randomized controlled trials (RCTs) and systematic reviews indicates that curcumin and ginger exhibit pronounced anti-inflammatory and antioxidant activities, effectively alleviating joint pain and stiffness [11,12,13]. Boswellic acids are derived from the resin of *Boswellia* plants and have a long history of use in treating inflammatory diseases. Boswellia has been shown to suppress inflammatory mediators and ameliorate synovitis [14]. Collagen and eggshell membrane contribute to cartilage repair and maintenance [15,16], while krill oil, which is rich in omega-3 polyunsaturated fatty acids, is thought to improve the joint microenvironment [17,18]. Further, vitamin D is believed to play a role in bone health and immune regulation [19].

Nevertheless, the available clinical evidence regarding the efficacy of these nutritional supplements in KOA primarily consists of studies comparing individual supplements to placebo, with a paucity of high-quality, head-to-head trials directly comparing different supplements. This evidence structure has limited conventional pairwise meta-analyses to evaluating the efficacy of each supplement versus placebo, making it difficult to systematically compare and rank the relative effectiveness of various supplements. Against this backdrop, network meta-analysis (NMA), an internationally recognized evidence synthesis methodology, would enable all supplements to be incorporated into a single evidence network using placebo as a common comparator, thereby integrating both direct and indirect comparison data. NMA not only compensates for the lack of direct evidence but also facilitates the ranking of multiple interventions according to their efficacy. Therefore, the present study employs NMA to systematically evaluate and compare the efficacy of seven commonly used nutritional supplements for KOA, aiming to provide clinicians and patients with more robust, comprehensive, and comparative evidence to inform management.

## 2. Materials and Methods

### 2.1. Data Sources and Searches

This study was registered in the International Prospective Register of Systematic Reviews database (Registration No. CRD420251044645), in accordance with the Preferred Reporting Items for Systematic Reviews Incorporating Network Meta-Analyses (Extension Statement) guidelines [20].

The search strategy includes eight core subject terms and their related free words, combined using appropriate Boolean operators (such as AND, OR) to ensure a comprehensive and targeted search. The specific subject terms are krill oil, curcumin, collagen, eggshell membrane, vitamin D, ginger, Boswellia, and knee osteoarthritis. The search formula is “Knee Osteoarthritis” AND (“Curcumin” [Mesh] OR “Vitamin D” [Mesh] OR “Krill” [Title/Abstract] OR “Eggshell” [Title/Abstract] OR “Boswellia” [Mesh] OR “Ginger” [Title/Abstract] OR “Collagen” [Mesh]) to identify relevant studies. The complete details of the search strategy are provided in Supplementary Material S1.

### 2.2. Study Selection

Two authors (Y.T.Z. and Y.F.G.) independently evaluated all obtained studies. After removing duplicates and screening the titles and abstracts, the full texts of potentially relevant studies were reviewed to identify eligible trials. In cases where disagreements arose during the evaluation process, they were resolved through discussion between the two authors, with any unresolved issues being referred to a third reviewer. This process was followed at each stage of the review, including during title/abstract screening and full-text assessment. Additionally, reference lists of relevant articles were examined to ensure that all appropriate papers were included.

The inclusion criteria were as follows: (1) study design: randomized controlled trials (RCTs); (2) participants: patients diagnosed with knee osteoarthritis (KOA) according to any recognized diagnostic criteria (e.g., ACR, clinical, or imaging-based diagnosis), and age > 18 years; (3) intervention: experimental group treated with nutritional supplements (eggshell membrane, vitamin D, Boswellia, curcumin, ginger, krill oil, collagen), and control group treated with placebo. There were no restrictions on dosage, formulation, or duration of intervention; (4) outcome measures: (a) Western Ontario and McMaster Universities Osteoarthritis Index (WOMAC), a commonly used scale for assessing symptoms and function in patients with knee or hip osteoarthritis, including pain (WOMAC pain), stiffness (WOMAC stiffness), and function (WOMAC function); (b) visual analog scale (VAS) for pain severity, typically ranging from 0 (no pain) to 10 (worst pain); (c) number of adverse events. Adverse events (AEs) are defined as any unfavorable health conditions that occur during a clinical study, potentially related to the intervention, and are assessed using subjective criteria (such as self-reported symptoms by the patient) and objective criteria (such as clinical examinations and laboratory test results). We excluded studies as follows: (1) Studies for which the full text could not be obtained after reasonable attempts (such as interlibrary loan or contacting authors) were excluded. (2) Studies that involved combination interventions—either multiple nutritional supplements used together or nutritional supplements combined with non-nutritional therapies—were excluded. Only studies evaluating a single nutritional supplement as a monotherapy compared to placebo were included. (3) Studies without eligible outcome measures were excluded. (4) Studies were excluded if they did not report sufficient data for quantitative synthesis of primary outcomes (such as means and standard deviations or other effect measures), or if key information regarding interventions, control groups, sample size, or outcome assessment was missing. (5) Non-English language publications were excluded.

### 2.3. Data Extraction and Outcome Measures

All data were extracted from the included studies by 2 reviewers using standardized data extraction forms. Methodological information included study characteristics (first author, country, year of publication, sample size, number of male and female participants, and intervention dosage), participant characteristics (age of patients and duration of KOA), as well as the interventions used in the experimental and control groups. Any disagreements were resolved through discussion with a third reviewer.

Although numerous outcome measures for KOA are in use, such as the Knee Injury and Osteoarthritis Outcome Score (KOOS), the Lequesne Index, and the Short Form-36 Health Survey (SF-36), this review focused specifically on the Western Ontario and McMaster Universities Osteoarthritis Index (WOMAC) and the visual analog scale (VAS) for pain. The primary rationale is that the WOMAC is an internationally recognized, highly reliable instrument for assessing pain, stiffness, and functional limitations in KOA patients, and has been widely adopted in clinical research [21]. In addition, the VAS is a standard and sensitive tool for quantifying pain intensity, with established validity and ease of administration, and is also frequently used in KOA studies [22]. Additionally, both WOMAC and VAS are well-suited for network meta-analysis, as their changes from baseline to post-intervention can be quantitatively compared across studies. Thus, selecting WOMAC and VAS as primary outcome measures ensures both the robustness of the findings and comparability across studies.

### 2.4. Quality Evaluation and Risk of Bias Assessment

Two reviewers independently assessed the risk of bias of the included studies using the Cochrane Risk of Bias Assessment Tool (version 5.3) with RevMan 5.4.1 statistical software [23]. The complete details of the search strategy are provided in Supplementary Material S2.

The evaluation covered the following domains: generation of the random sequence, allocation concealment, blinding of participants and personnel, blinding of outcome assessors, completeness of outcome data, selective reporting, and other potential sources of bias. After completing the assessments, the results were cross-checked. Any disagreements were resolved through discussion with a third reviewer.

### 2.5. Data Synthesis and Statistical Analysis

All evidence networks for each outcome were constructed using R software (version 4.5.0). For outcomes with at least ten studies, publication bias was assessed by conducting Egger’s test and generating comparison-adjusted funnel plots [24]. All outcomes in this study were analyzed as continuous variables based on the change from baseline to post-intervention, which facilitates a consistent evaluation of treatment effects across studies. With regard to outcome measures, the Western Ontario and McMaster Universities Osteoarthritis Index (WOMAC) and visual analog scale (VAS) were selected as the primary endpoints and analyzed separately. As all included studies reported WOMAC using the 0–96 scale and VAS using the 0–10 scale, mean difference (MD) was uniformly adopted as the effect size, enhancing both the interpretability and comparability of the results.

A Bayesian random-effect model was employed to conduct the NMA. NMA allows for the integration of both direct and indirect evidence, enabling simultaneous comparison and probabilistic ranking of multiple interventions—even in the absence of direct head-to-head trials. The ranking of interventions was determined by the surface under the cumulative ranking curve (SUCRA), which quantifies the probability that each intervention is the most effective. Compared to traditional pairwise meta-analysis, NMA offers a more comprehensive synthesis of evidence, providing valuable guidance for clinical decision-making, particularly when direct comparisons are limited.

It is noteworthy that the majority of the studies included in this analysis compared a single supplement with placebo, with few direct head-to-head trials between supplements, resulting in a “star-shaped” network structure. Although conventional meta-analysis or subgroup analysis could theoretically be used, NMA enables a unified comparison and ranking of all supplements, thus providing more robust and systematic evidence for clinical practice and effectively addressing the limitations posed by the lack of direct comparisons.

A Markov Chain Monte Carlo (MCMC) random-effect model was applied, with four chains simulated, a tuning iteration of 20,000, a simulation iteration of 50,000, a thinning interval of 10, and an inference sample size of 10,000. The potential scale reduction factor (PSRF) was used to evaluate model convergence, with values approaching 1 indicating good convergence and reliable results from the consistency model. As no closed loops were formed in this study, inconsistency testing was not performed.

## 3. Results

### 3.1. Literature Search and Screening

**Figure 1:**
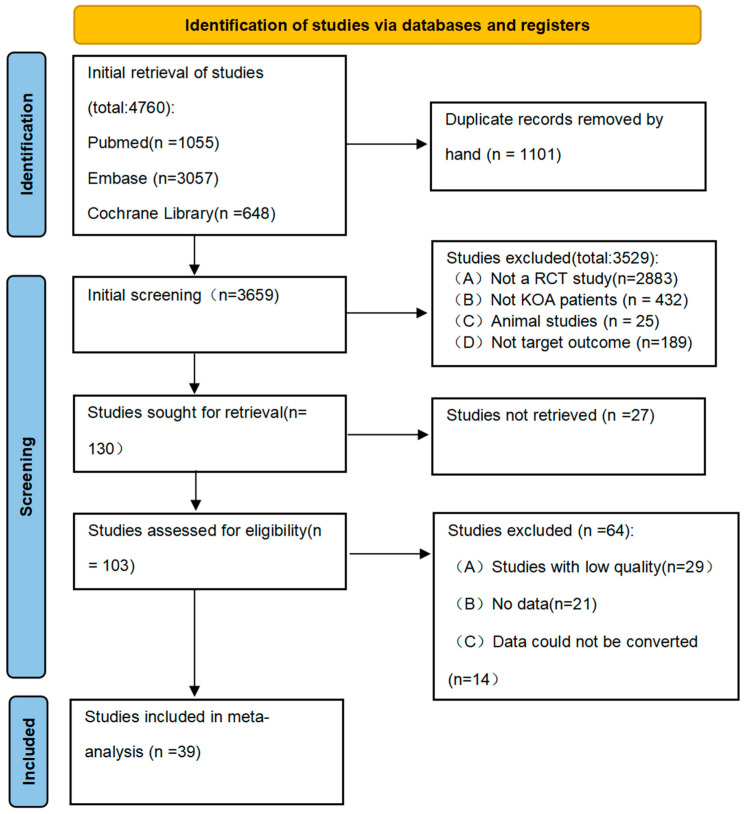
Preferred Reporting Items for Systematic reviews and Meta-Analysis (PRISMA) diagram.

Our literature search yielded 4760 potentially relevant records. After removal of duplicates, the titles and abstracts of 3659 records were screened, and the full texts of 130 articles were evaluated. Ultimately, 39 studies [18,19,25,26,27,28,29,30,31,32,33,34,35,36,37,38,39,40,41,42,43,44,45,46,47,48,49,50,51,52,53,54,55,56,57,58,59,60,61] were judged to be eligible for inclusion in this meta-analysis. The detailed selection process for including these studies is presented in Figure 1.

### 3.2. Quality Assessment of the Included Studies

**Figure 2:**
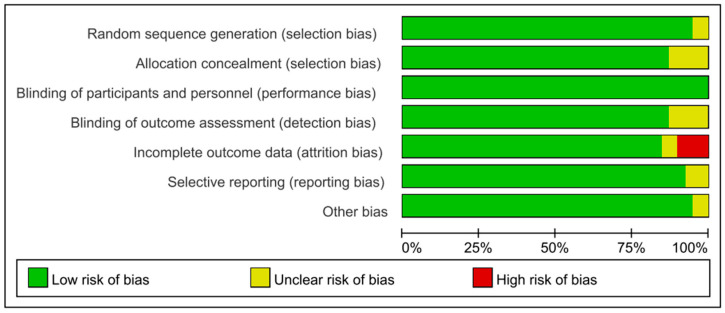
Risk of bias summary plot.

Regarding the method of random sequence generation, 37 studies explicitly reported the use of a random number table and were assessed as having a low risk of bias, while 2 studies [53,54] only mentioned randomization without specifying the method and were rated as unclear risk. For allocation concealment, five studies did not provide explicit details and were therefore judged as unclear risk. All studies were considered low risk for blinding of participants and personnel. With respect to blinding of outcome assessors, five studies did not provide relevant information and were assessed as unclear risk. For completeness of outcome data, four studies [18,25,32,38] were rated as high-risk due to missing data, while the remainder were deemed low-risk. In terms of selective reporting, studies that did not provide a clinical trial registration number (e.g., ClinicalTrials.gov) or a publicly available pre-specified study protocol were considered unclear risk. For other sources of bias, all studies failed to report information such as funding sources and were therefore rated as unclear risk. Overall, the included studies were of moderate to high methodological quality. Most studies demonstrated a low risk of bias in random sequence generation, blinding, and completeness of outcome data. However, some studies lacked detailed reporting on allocation concealment, blinding of outcome assessors, selective reporting, and other potential sources of bias, leading to certain domains being rated as unclear or high-risk. These limitations may introduce some uncertainty into the interpretation of specific outcomes. Nonetheless, the overall quality of the included literature was acceptable and provides a relatively robust foundation for the evidence synthesis and interpretation of results in this review. Details are shown in Figure 2. The complete details of the search strategy are provided in Supplementary Material S3.

### 3.3. Characteristics of the Eligible Studies

The included studies were published between 2001 and 2024, comprising 39 articles and 42 independent studies with a total of 4599 patients. Although there were variations in age, sex distribution, and intervention duration, all patients included in these studies were diagnosed with knee osteoarthritis. The duration of interventions ranged from 4 weeks as the shortest [53] to 36 months as the longest [59] Dosages of interventions also varied widely and were recorded using the commercial names of the extracts. The proportion of female patients was significantly higher than that of males, which may be attributed to the greater susceptibility of elderly women to osteoarthritic degeneration. The primary outcome measures were pain scores and joint function scores. Prior to literature retrieval, this study prospectively defined seven common and representative nutritional supplements, including eggshell membrane (five studies [25,26,27,28,29]), curcumin (eight studies [30,31,32,33,34,35,36,37]), collagen (five studies [38,39,40,41,42]), krill oil (four studies [18,43,44,45]), Boswellia (eight studies [36,46,47,48,49,50,51,52], ginger (six studies [53,54,55,56,57,58]), and vitamin D (four studies [19,59,60,61]). The main characteristics of the included studies are summarized in Table 1.

### 3.4. Results from Network Meta-Analysis

**Figure 3:**
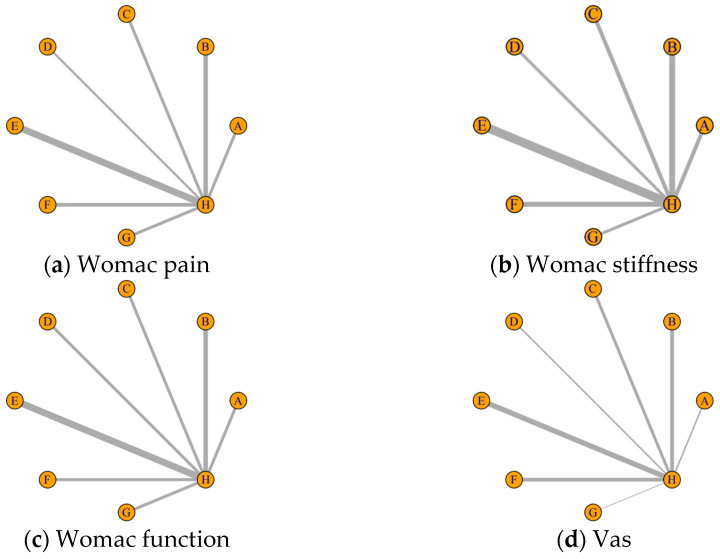
Network evidence plot. A: Eggshell membrane; B: curcumin; C: collagen; D: krill oil; E: Boswellia; F: ginger; G: vitamin D; H: placebo.

To evaluate the efficacy of the seven nutritional supplements on various outcome measures in patients with knee osteoarthritis, we constructed an intervention network comprising seven competing treatments (see Figure 3). For WOMAC Pain, 35 studies involving 7 nutritional supplements were included, resulting in seven direct comparisons. For WOMAC Stiffness, 34 studies with 7 supplements formed seven direct comparisons. For WOMAC Function, 35 studies and 7 supplements also resulted in seven direct comparisons. For the VAS outcome, 26 studies involving 7 supplements yielded seven direct comparisons. In the evidence networks for all outcome measures, no closed loops were formed. In the network plots, the size of each node represents the sample size for each intervention, while the thickness of the connecting lines indicates the number of RCTs for each comparison.

In the WOMAC pain score analysis, 35 studies involving 4015 patients and 7 nutritional supplements were included. The results indicated that only Boswellia demonstrated a statistically significant effect in terms of pain relief (MD = 10.58, *p* < 0.05). While other supplements (curcumin, ginger, vitamin D, krill oil, eggshell membrane, and collagen) showed greater efficacy than placebo, none achieved statistical significance (*p* > 0.05), as shown in Table 2.

In the WOMAC stiffness score analysis, 34 studies involving 3868 patients and 7 nutritional supplements were included. The results showed that only Boswellia demonstrated a statistically significant improvement in stiffness (MD = 9.47, *p* < 0.05). Although other supplements, including krill oil, ginger, collagen, vitamin D, eggshell membrane, and curcumin, showed greater effects than placebo, none reached statistical significance (*p* > 0.05), as shown in Table 3.

In the WOMAC function score analysis, a total of 35 studies involving 4253 patients and 7 nutritional supplements were included. The results showed that krill oil (MD = 14.01, *p* < 0.05), Boswellia (MD = 14.00, *p* < 0.05), curcumin (MD = 9.96, *p* < 0.05), and collagen (MD = 9.42, *p* < 0.05) demonstrated statistically significant improvements in function. Other interventions, although showing greater effect sizes than placebo, did not reach statistical significance (*p* > 0.05), as shown in Table 4.

In the analysis of VAS scores, a total of 26 studies involving 2753 patients and 7 different nutritional supplements were included. The results showed that Boswellia (MD = 17.26, 95% CI: 8.06 to 26.52, *p* < 0.05), collagen (MD = 16.65, 95% CI: 4.32 to 29.09, *p* < 0.05), curcumin (MD = 12.34, 95% CI: 1.59 to 23.34, *p* < 0.05), and ginger (MD = 11.89, 95% CI: 1.01 to 22.49, *p* < 0.05) were associated with statistically significant reductions in pain. These results demonstrate not only statistical significance but also clinical relevance, as the observed changes in VAS scores (MD ranging from 11.89 to 17.26) represent meaningful reductions in pain levels. A reduction in pain of 1–2 points on a 0–10 scale is typically considered clinically significant. Therefore, the changes observed in this analysis suggest substantial improvements in pain perception, which could substantially affect patients’ quality of life. In contrast, other interventions, although showing greater effect sizes than placebo, did not reach statistical significance (*p* > 0.05), indicating their effects may not be consistent enough for clinical recommendation, as shown in Table 5.

### 3.5. Adverse Events

In the 41 studies reporting adverse events, significant inconsistencies were observed in the reporting practices. Specifically, 8 studies explicitly stated that no significant adverse events occurred in either group; 6 studies did not specify the number or types of adverse events; 22 studies provided detailed reports on adverse events but clearly indicated that these events were unrelated to the interventions; and only 5 studies specifically reported adverse events that were potentially associated with the interventions (as shown in Table 6). These discrepancies highlight substantial variability in the reporting practices and standards across studies, which may impact the comprehensive evaluation of the safety and efficacy of the interventions. Therefore, future studies should adopt more standardized reporting of adverse events to ensure data comparability and the reliability of conclusions.

### 3.6. Ranking of Interventions

**Figure 4:**
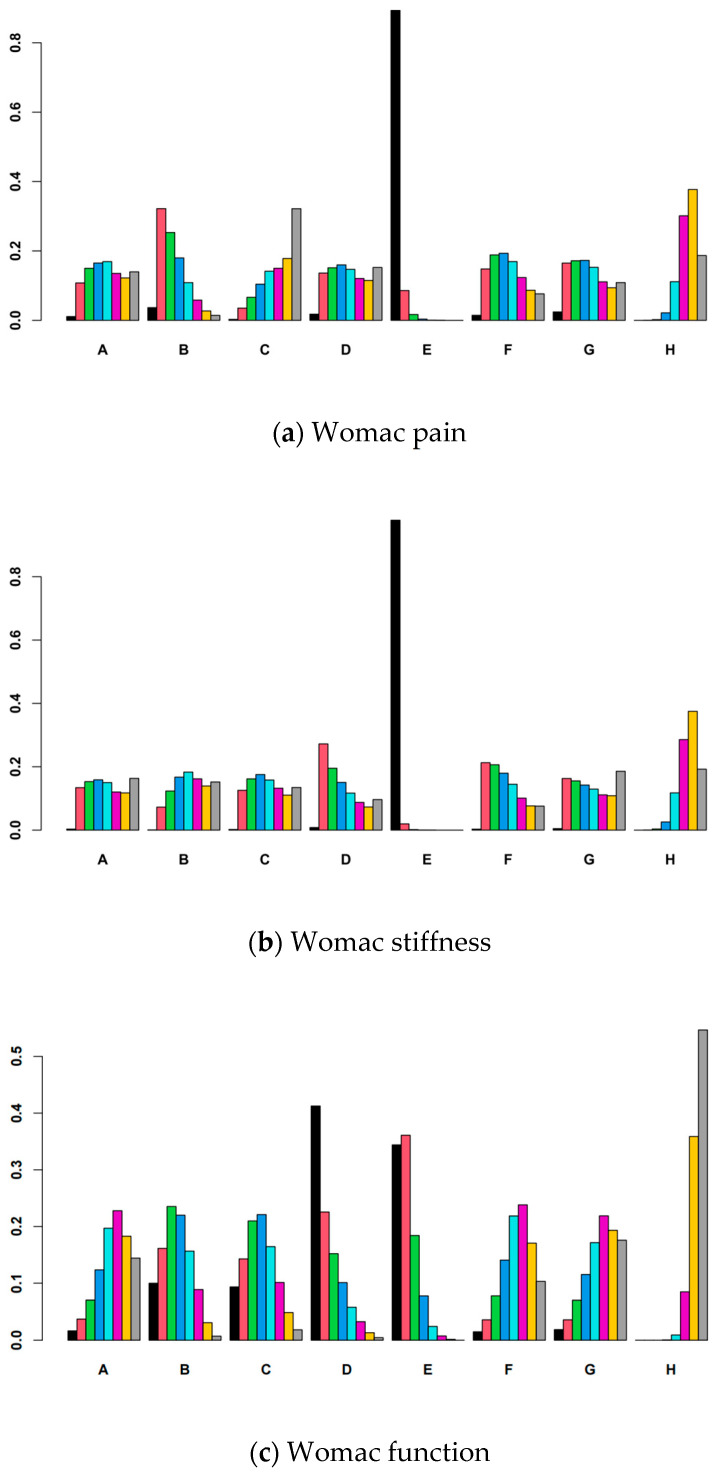

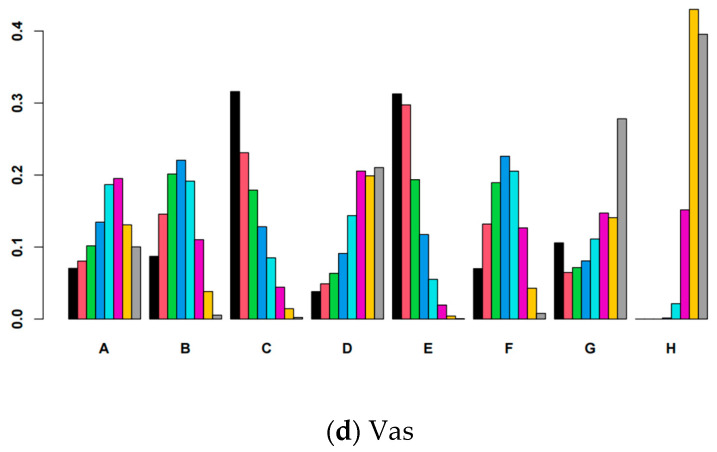
Probability ranking plots for each outcome measure (A = eggshell membrane; B = curcumin; C = collagen; D = krill oil; E = Boswellia; F = ginger; G = vitamin D; H = placebo).

Bayesian statistical methods were used to generate probability rankings for the seven nutritional supplements. For each outcome measure, a Rank 1 probability indicates that the intervention is most likely to be the optimal treatment for that specific outcome. For the indicators WOMAC pain, WOMAC stiffness, WOMAC function, and VAS, a higher Rank 1 probability value suggests superior efficacy of the intervention for that outcome, as shown in Figure 4.

For WOMAC pain, the ranking based on SUCRA values from highest to lowest was as follows: Boswellia (0.981) > curcumin (0.663) > ginger (0.503) > vitamin D (0.498) > krill oil (0.450) > eggshell membrane (0.432) > collagen (0.273). Boswellia showed the highest SUCRA value, indicating that this supplement has the highest probability of being ranked as the best option for pain relief among all interventions. The complete details of the search strategy are provided in Supplementary Material S4.

For WOMAC stiffness, the SUCRA-based ranking was as follows: Boswellia (0.997) > krill oil (0.553) > ginger (0.537) > collagen (0.447) > vitamin D (0.439) > eggshell membrane (0.434) > curcumin (0.391). Again, Boswellia had the highest SUCRA value, indicating the greatest probability of being ranked as the most favorable intervention for alleviating stiffness.

Regarding WOMAC function, the SUCRA-based ranking was Boswellia (0.842) > krill oil (0.808) > curcumin (0.629) > collagen (0.598) > ginger (0.368) > eggshell membrane (0.345) > vitamin D (0.329). Notably, Boswellia and krill oil had substantially higher SUCRA values than the other interventions, suggesting a greater probability of being ranked as the most favorable options for improving joint function.

For VAS scores, the ranking was Boswellia (0.803) > collagen (0.766) > curcumin (0.601) > ginger (0.578) > eggshell membrane (0.443) > vitamin D (0.368) > krill oil (0.326). Boswellia and collagen exhibited relatively high SUCRA values, suggesting a potential advantage in reducing pain as measured by the visual analog scale.

### 3.7. Publication Bias

**Figure 5:**
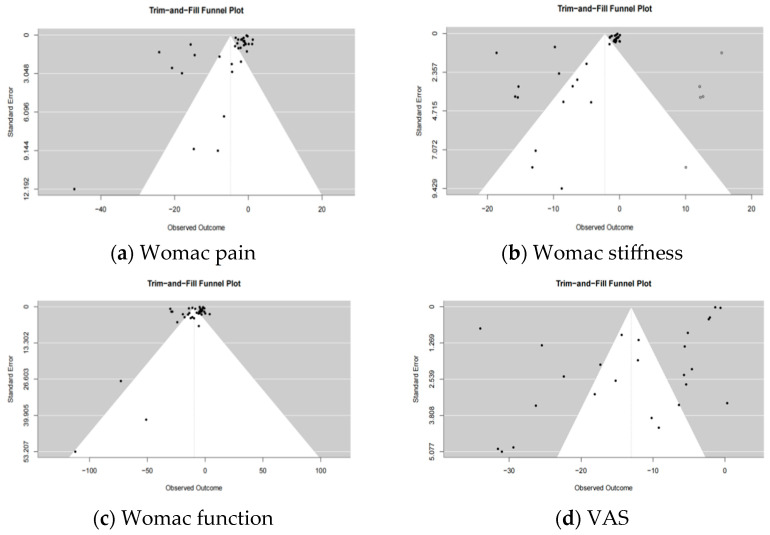
Adjusted funnel plots.

Comparison-adjusted funnel plots were generated for each outcome with more than 10 studies—WOMAC pain, WOMAC stiffness, WOMAC function, and VAS—with the effect size on the x-axis and the standard error of the effect size on the y-axis. The plots revealed poor symmetry around the zero line for all outcomes, indicating a high likelihood of publication bias or small-study effects, as shown in Figure 5.

### 3.8. Convergence Assessment

For the outcome measures of WOMAC pain, WOMAC stiffness, WOMAC function, and VAS, the PSRF values were all close to 1, indicating good convergence and reliable results from the consistency model.

### 3.9. Assessment of Inconsistency

For all outcome measures, no closed loops were formed between the interventions in the experimental groups and, therefore, inconsistency tests were not conducted.

## 4. Discussion

Knee osteoarthritis (KOA) causes significant damage to the tissue in the knee joint [62]. As articular cartilage gradually degenerates, mechanical loads are transmitted directly to the subchondral bone, resulting in bone sclerosis, microstructural disruption, and osteophyte formation [63]. The inflammatory response induces the sustained release of pro-inflammatory cytokines such as IL-1β and TNF-α, which activate degradative enzymes, accelerate cartilage matrix breakdown, and lead to abnormal bone remodeling [64]. These processes also affect the synovium and surrounding tissues, causing synovial hyperplasia and further osteophyte formation, thereby exacerbating joint structural damage [65]. Such pathological changes ultimately lead to joint space narrowing, exposure of subchondral bone, persistent pain, limited mobility, joint stiffness, and abnormal gait [66]. As the disease progresses, joint stability declines, increasing the risk of walking difficulties and falls [67]. Persistent pain and functional limitations markedly diminish patients’ quality of life and may predispose individuals to psychological comorbidities, including anxiety and depression [68].

Current treatments for KOA are mainly divided into non-pharmacological, pharmacological, and surgical approaches. Non-pharmacological interventions, encompassing weight management, physiotherapy, and structured exercise programs, are generally regarded as safe; however, a subset of patients may experience exacerbation of joint discomfort or sustain injuries during rehabilitation [69,70]. Pharmacological treatments—including non-steroidal anti-inflammatory drugs (NSAIDs), hyaluronic acid injections, and chondroprotective agents—can cause gastrointestinal reactions, cardiovascular events, and renal impairment. Intra-articular corticosteroid injections also carry the risk of infection, local tissue atrophy, and cartilage damage [71]. Surgical treatments, such as arthroscopic debridement and total knee arthroplasty, can effectively alleviate symptoms, but are associated with risks including infection, thrombosis, and subsequent prosthesis loosening [72].

In recent years, a growing body of clinical and basic research has demonstrated that nutritional supplements, due to their multi-target effects, low side effect profiles, and good patient compliance, have become an important adjunct in the management of KOA [73]. These supplements not only provide essential micronutrients, but also improve chronic disease outcomes through anti-inflammatory, antioxidant, immunomodulatory, and tissue repair mechanisms [74,75]. Therefore, appropriate nutraceutical supplementation is expected to become an important component of comprehensive management for chronic diseases such as KOA, providing new options for long-term patient care [73].

With respect to safety, among the seven studies reporting adverse events related to the interventions, two involved eggshell membrane, one involved curcumin, one involved collagen, one involved ginger, and two involved vitamin D. All reported adverse events were mild and resolved after appropriate management. However, since contraindications for some nutritional supplements remain unclear, patients should be carefully monitored for adverse reactions following administration. In the event of adverse reactions, it is recommended to discontinue the supplement and monitor and manage the patient’s clinical condition.

It is also worth noting that some herbal supplements, especially those with potent pharmacological properties, may cause liver damage, particularly when used in combination with other medications. Studies have shown that high-dose treatment of mice with curcumin-loaded nanocomposites led to some degree of liver damage [76]. As the primary detoxification organ, the liver is responsible for the metabolism of many herbal components. Therefore, these substances may affect liver function or interact with other drugs, increasing the risk of liver damage. Such interactions may alter the metabolic rate of herbal components or medications, thereby changing their effects, increasing toxicity, and potentially harming liver health. Therefore, it is always advisable to consult a medical practitioner when patients are on medications, to ensure safety and prevent possible risks.

While the optimal intervention varied across different outcome measures, further analysis revealed that Boswellia consistently ranked first for WOMAC pain, stiffness, function, and VAS scores. Curcumin was among the top three for improvement in WOMAC pain, function, and VAS, while curcumin and ginger both ranked among the top three for WOMAC pain and stiffness. Krill oil demonstrated excellent performance in the improvement of stiffness. In addition, collagen ranked second for VAS scores. It is important to note that studies supporting Boswellia’s superiority rely heavily on weaker evidence. While some studies suggest Boswellia is effective in alleviating pain and improving stiffness, function, and VAS scores, the quality of the evidence is still lacking. Many studies are limited by small sample sizes, design biases, participant heterogeneity, and insufficient statistical analysis. Therefore, results should be interpreted cautiously, and future research should improve randomized controlled trial designs and sample sizes to ensure the reliability and validity of findings.

Boswellic acids are resin extracts from plants of the genus Boswellia (family Burseraceae), also known as frankincense or guggul, and have been traditionally used in Ayurvedic medicine to treat inflammatory conditions, including osteoarthritis (OA) [76]. These extracts contain a variety of bioactive components, among which 3-acetyl-11-keto-β-boswellic acid (AKBA), 11-keto-β-boswellic acid (KBA), and β-boswellic acid (BA) have attracted particular attention due to their significant bioactivity in vitro and in vivo [77]. Boswellic acids exert their anti-inflammatory effects primarily by inhibiting 5-lipoxygenase (5-LOX) and cyclooxygenase (COX)-mediated prostaglandin synthesis, as well as modulating the immune system, thereby enhancing their anti-inflammatory and therapeutic potential [78]. Bannuru et al. were among the first to evaluate the effects of curcumin and Boswellia in 2018 and suggest that Boswellia may be effective for improving pain and function in knee osteoarthritis. However, their review included only four direct comparison trials of Boswellia versus placebo, and in one of these, WOMAC was not the outcome measure [79]. In a review by Yu et al., it was recommended that Boswellia and its extracts be administered at a dose of 100–250 mg for at least 4 weeks [80]. However, four of the included studies used combinations with other bioactive molecules, and two used active (non-placebo) control groups; thus, only three studies were available to evaluate the effect of Boswellia alone compared to placebo, which was insufficient to confirm its independent efficacy [80]. A review by Thomas Dalmonte et al. provided a more comprehensive summary of placebo-controlled trials, concluding that Boswellia resin extracts, as a complementary and alternative medicine (CAM) modality, have a positive effect on symptoms of knee osteoarthritis, especially for patients who cannot tolerate NSAIDs [14].

Curcumin, an active compound extracted from the rhizome of Curcuma longa (turmeric), possesses a wide range of biological activities, including anti-inflammatory, antioxidant, and anticancer properties [81]. Curcumin and its derivatives exert their effects by inhibiting the NF-κB signaling pathway, which downregulates pro-inflammatory factors such as COX-2, JNK, PI3K, and AP-1, thereby reducing the secretion of pro-inflammatory mediators like IL-6, IL-1β, TNF-α, and alleviating joint inflammation [82,83]. Two meta-analyses have compared the efficacy of curcumin and its extracts with NSAIDs and placebo, demonstrating that curcumin offers therapeutic effects comparable to NSAIDs, while providing a better safety profile [11,84]. However, further research has shown that curcumin has relatively low bioavailability, necessitating strategies to enhance its effectiveness [85]. For instance, Mahtab Baharizade et al. developed a hybrid system (SNE-POG) that combines physically cross-linked PEG-based organogels with a self-nanoemulsifying drug delivery system (SNEDDS), which significantly improves curcumin’s solubility and bioavailability [86]. Additionally, Liuting Zeng’s study found that continuous use of turmeric extract and curcumin supplements for over 12 weeks resulted in better outcomes in knee osteoarthritis (KOA) patients, although the optimal duration still requires further investigation [87]. Overall, curcumin shows significant benefits in the treatment of KOA, with comparable efficacy between high and low doses; however, the optimal dosing regimen remains to be determined [11,82,84].

Ginger (Zingiber officinale), a perennial herb of the Zingiberaceae family, has a long history of medicinal use for the treatment of various conditions. Ginger contains a rich array of bioactive compounds, such as gingerols, shogaols, paradols, and terpenoids, which contribute to its various biological activities. [88]. In addition to antioxidant and anti-inflammatory effects, ginger possesses analgesic, antipyretic, and antimicrobial properties, all of which are considered important in the management of KOA [89,90]. Previous studies have shown that ginger can inhibit the production of inflammatory mediators—including nitric oxide (NO) and prostaglandin E2 (PGE2)—in porcine chondrocytes, suggesting a positive cellular effect on KOA [91]. A review by Bartels et al. indicated that ginger is superior to placebo for relieving OA pain and disability, with no significant serious adverse events reported [12]. The analgesic effect of ginger is likely mediated by multiple mechanisms, such as inhibition of prostaglandin synthesis via COX and LOX pathways, antioxidant activity, inhibition of the NF-κB transcription factor, and effects on vanilloid pain receptors [92]. However, the pungency of ginger can limit its consumption. To address this, processing methods such as steaming, fermentation, aging, roasting, and preparation of koji-ginger have been developed to prolong shelf life, enhance the content of bioactive compounds, and improve safety [93]. Notably, a recent randomized trial confirmed both the efficacy and safety of steamed ginger extract in patients with mild osteoarthritis [58].

Krill oil is rich in omega-3 polyunsaturated fatty acids, especially eicosapentaenoic acid (EPA) and docosahexaenoic acid (DHA), as well as astaxanthin, a potent antioxidant [94]. Studies indicate that krill oil offers multiple benefits in the treatment of KOA. Its primary mechanisms of effect include inhibition of inflammatory signaling pathways such as NF-κB, leading to reduced levels of pro-inflammatory factors (e.g., IL-1β, TNF-α, and CRP) in synovial fluid and cartilage, thereby attenuating joint inflammation [95]. Furthermore, EPA and DHA in krill oil help regulate lipid metabolism, reduce the production of inflammatory mediators such as prostaglandin E2, and protect chondrocytes from oxidative stress-induced damage through antioxidant effects [96]. Clinical studies have shown that krill oil supplementation significantly alleviates joint pain and improves joint function in KOA patients, with good safety and gastrointestinal tolerance. Overall, krill oil is a promising adjunctive therapy for the management of KOA [97].

Collagen is a vital biopolymer that provides structural support and elasticity to joint cartilage, playing a key role in maintaining cartilage integrity and function by absorbing mechanical shock and reducing friction [98]. However, native collagen exhibits low bioavailability due to poor absorption, thus only hydrolyzed collagen can serve as a physiologically effective supplement [99]. Undenatured type II collagen, primarily derived from chicken sternum cartilage, is believed to modulate both humoral and cellular immune responses. It exerts a protective effect against joint damage by inducing and recruiting regulatory T cells (Tregs), which in turn promote the secretion of anti-inflammatory cytokines [100,101]. Furthermore, Tregs can stimulate chondrocytes to synthesize extracellular matrix components through the release of these cytokines, contributing to cartilage repair and maintenance [102,103]. Recent reviews indicate that oral collagen supplementation significantly alleviates symptoms of osteoarthritis (OA), as evidenced by reductions in total WOMAC and visual analog scale (VAS) scores. However, the efficacy of collagen on specific WOMAC subscales—such as pain and physical function—remains a subject of ongoing debate [15]. Current evidence suggests that collagen supplements hold potential as an adjunctive therapy for alleviating osteoarthritis symptoms.

In this study, we included different dosages and pharmaceutical forms of the same supplement (such as curcumin supplements, including Turmacin™, C3 Complex^®^, Haridra^®^, etc.). This approach was taken because research on these nutritional supplements is still evolving, and no unified standard has been established. Using different dosages and pharmaceutical forms of supplements allows us to more comprehensively assess their potential effects in the treatment of knee osteoarthritis. However, it is important to note that different pharmaceutical forms and dosages may have varying effects on efficacy. For example, the bioavailability of curcumin can vary significantly depending on the pharmaceutical form, which may lead to an inability to fully control the impact of these variations when comparing different supplements. Furthermore, different dosages of supplements may influence the duration and intensity of their effects, leading to some heterogeneity in the study. These factors may affect our comprehensive evaluation of the supplement’s effectiveness, and the comparison of different dosages and forms may introduce some uncertainty in the interpretation of the final results.

### 4.1. Study Strengthens and Limitations

Currently, research on the above nutritional supplements is still in the exploratory stage, and there are no authoritative guidelines to guide their recommended level in the treatment of knee osteoarthritis (KOA). Most clinical studies at this stage focus on evaluating their therapeutic effects, and more research is needed to reach a definitive consensus and shift.

Additionally, several limitations should be noted. First, there was considerable heterogeneity among the included studies. For some outcomes, the number of relevant studies was limited, and the distribution of literature across different supplements for the same outcome was uneven, a fact which may affect the robustness of the results. Second, the treatment duration, dosage, and formulation of nutritional supplements varied and lacked standardization; future studies should aim to unify and standardize intervention protocols. Third, there were relatively few studies assessing VAS pain scores, highlighting the need for more frequent use of VAS in evaluating KOA severity in future research. In addition, there is a lack of direct head-to-head comparisons between different supplements, which may affect the reliability of efficacy rankings. Finally, follow-up periods in some studies were relatively short, limiting the ability to fully assess the long-term efficacy and safety of these interventions. Therefore, there is an urgent need for high-quality, multicenter, large-sample, and long-term randomized controlled trials to provide more robust evidence regarding the application of nutritional supplements in the management of knee osteoarthritis.

### 4.2. Conclusions

Compared to placebo, nutritional supplements may improve symptoms in patients with knee osteoarthritis (KOA) by alleviating WOMAC pain, stiffness, and function scores, as well as the VAS pain index, without increasing the incidence of adverse events. The results indicate that certain nutritional supplements—particularly Boswellia, curcumin, collagen, ginger, and krill oil—offer potential benefits in symptom relief and functional improvement. Notably, based on indirect and limited head-to-head comparisons, Boswellia exhibited the highest probability of being the optimal intervention across multiple outcome measures. The favorable safety profiles of these supplements further support their role as important components of non-pharmacological management strategies for KOA, especially for patients who are intolerant of, or have contraindications to, conventional pharmacological or surgical treatments. Nevertheless, large-scale, high-quality randomized controlled trials are still needed to provide more robust evidence.

## Figures and Tables

**Table 1:** Characteristics of the publications included in the meta-analysis.

Study	Country Year	N	Treatment	Control	Treatment Duration	Age (Years)		Female	Dosage	Outcome
						T	C			
Ruff et al. [25]	USA 2009	60	Eggshell	Placebo	60 days	NR	NR	NR	NEM^®^ 500 mg/day	WOMAC
Cánovas et al. [26]	Spain 2022	51	Eggshell	Placebo	8 weeks	36.36 ± 13.54	41.31 ± 14.36	27/51	ESM^®^ 500 mg/day	VAS
Hewlings et al. [27]	USA 2019	88	Eggshell	Placebo	12 weeks	NR	NR	63/88	BiovaFlex^®^ 450 mg/day	WOMAC
Eskiyurt et al. [28]	Turkey 2019	166	Eggshell	Placebo	90 days	55.9 ± 11.9	58.5 ± 9.7	134/166	NEM^®^ 500 mg/day	WOMAC
Park et al. [29]	Korea 2024	99	Eggshell	Placebo	12 weeks	57.73 ± 7.75	58.54 ± 8.28	79/99	NEM^®^ 500 mg/day	WOMAC; VAS
Hashemzadeh et al. [30]	Iran 2024	71	Curcumin	Placebo	6 weeks	54.11 ± 5.80	56.54 ± 5.77	60/71	SinaCurcumin™ 80 mg/day	WOMAC
Madhu et al. [31]	India 2013	60	Curcumin	Placebo	6 weeks	56.63 ± 10.58	56.77 ± 9.98	34/60	Turmacin™ 1000 mg/day	VAS
Panahi et al. [32]	Iran 2014	40	Curcumin	Placebo	6 weeks	57.32 ± 8.78	57.57 ± 9.05	31/40	C3 Complex^®^ 1500 mg/day	WOMAC; VAS
Srivastava et al. [33]	India 2016	160	Curcumin	Placebo	16 weeks	50.23 ± 8.08	50.27 ± 8.63	103/160	Haridra^®^ 1000 mg/day	WOMAC; VAS
Wang et al. [34]	Australia 2020	70	Curcumin	Placebo	12 weeks	61.3 ± 8.5	62.4 ± 8.8	39/70	Turmacin™ 1000 mg/day	WOMAC; VAS
Atabaki et al. [35]	Iran 2020	30	Curcumin	Placebo	12 weeks	49.13 ± 8.87	48.26 ± 7.81	30/30	SinaCurcumin^®^ 80 mg/day	VAS
Haroyan et al. [36]	Armenia 2018	134	Curcumin	Placebo	12 weeks	54.65 ± 8.84	56.04 ± 8.55	127/134	CuraMed^®^ 1500 mg	WOMAC
Panda et al. [37]	India 2018	50	Curcumin	Placebo	12 weeks	55.20 ± 8.58	53.12 ± 8.25	NR	Curene^®^ 500 mg	WOMAC; VAS
Benito-Ruiz et al. [38]	Spain 2009	207	Collagen	Placebo	24 weeks	58.7 ± 10.4	59.1 ± 11.6	192/207	Colnatur^®^ 10 g/day	WOMAC; VAS
Kumar et al. [39]	India 2014	60	Collagen	Placebo	13 weeks	NR	NR	BCP:18/30 PCP:27/30	Pork Collagen Peptide 10 g/day Bovine Collagen Peptide 10 g/day	VAS
Lugo et al. [40]	USA 2016	121	Collagen	Placebo	24 weeks	53.5 ± 7.9	53.1 ± 7.8	60/121	UC-II^®^ 40 mg/day	WOMAC; VAS
McAllindon et al. [41]	USA 2011	30	Collagen	Placebo	48 weeks	58.9 ± 8.0	60.3 ± 8.5	18/30	Fortigel^®^ 10 g/day	WOMAC
Schauss et al. [42]	USA 2012	88	Collagen	Placebo	10 weeks	54.3 ± 8.7	54.5 ± 9.8	41/88	BioCell Collagen^®^ 2000 mg/day	WOMAC
Stonehouse et al. [43]	Australia 2022	235	Krill oil	Placebo	24 weeks	59.9 ± 6.3	59.3 ± 6.6	77/235	Superba Boost™ 4 g/day	WOMAC
Laslet et al. [44]	Australian 2024	262	Krill oil	Placebo	24 weeks	61.7 ± 9.3	61.4 ± 9.9	122/262	krill oil softgel 2 g/day	WOMAC; VAS
Hill et al. [18]	Korean 2023	75	Krill oil	Placebo	12 weeks	57.0 ± 10.28	59.0 ± 11.82	44/75	FlexPro MD^®^ 600 mg/day	WOMAC; VAS
Deutsch et al. [45]	Canada 2007	90	Krill oil	Placebo	30 days	54.6 ± 14.8	55.3 ± 14.3	43/90	NKO™ 300 mg/day	WOMAC
Karlapudi et al. [46]	India 2018	70	Boswellia	Placebo	90 days	48.7 ± 1.13	50.3 ± 1.34	43/70	LI73014F2 400 mg/day	WOMAC; VAS
Karlapudi et al. [47]	India2021	67	Boswellia	Placebo	30 days	51.60 ± 8.48	51.81 ± 7.21	50/67	Aflapin^®^ 100 mg/day	WOMAC; VAS
Sengupta et al. [48]	India 2008	46	Boswellia	Placebo	90 days	53.22 ± 8.73	52.43 ± 9.65	33/46	5-Loxin^®^ 250 mg/day	WOMAC; VAS
Kumar et al. [49]	India 2024	80	Boswellia	Placebo	180 days	48.60 ± 7.39	47.93 ± 7.89	47/80	Aflapin^®^ 100 mg/day	WOMAC; VAS
Sengupta et al. [50]	India 2010	76	Boswellia	Placebo	90 days	Aflapin^®^; 53.2 ± 7.9 5-Loxin^®^ 51.6 ± 9.9	52.4 ± 7.5	Aflapin^®^; 22/38 5-Loxin^®^; 26/38	5-Loxin^®^ 100 mg/day Aflapin^®^ 100 mg/day	WOMAC; VAS
Vishal et al. [51]	India 2011	59	Boswellia	Placebo	30 days	53.2 ± 6.5	55.3 ± 8.8	37/59	Aflapin^®^ 100 mg/day	WOMAC VAS
Haroyan et al. [36]	Armenia 2018	135	Boswellia	Placebo	12 weeks	57.91 ± 9.02	56.04 ± 8.55	127/135	Curamin^®^ 1500 mg/day	WOMAC
Majeed et al. [52]	India 2019	48	Boswellia	Placebo	120 days	NR	NR	31/48	Boswellin^®^ 338.66 mg/day	WOMAC; VAS
Haghighi et al. [53]	Iran 2005	80	Ginger	Placebo	4 weeks	58.3 ± 0.33	58.4 ± 0.36	23/80	Zingiber officinale 30 mg/day	VAS
Zakeri et al. [54]	Iran 2011	204	Ginger	Placebo	6 weeks	48.4 ± 11.1	45.74 ± 12.5	164/204	Zintoma^®^ 500 mg/day	WOMAC; VAS
Altman et al. [55]	USA 2001	247	Ginger	Placebo	6 weeks	64.0 6 11.5	66.3 6 11.6	152/247	EV.EXT 77 510 mg/day	WOMAC; VAS
Wigler et al. [56]	Israel 2003	29	Ginger	Placebo	12 weeks	64.7 (47–85)	59.3 (42–81)	23/29	Zintona EC^®^ 1000 mg/day	WOMAC
Afshar et al. [57]	Iran 2022	43	Ginger	Placebo	12 weeks	55.62 ± 8.646	54.86 ± 6.63	29/43	G-Rup^®^ 60 mL/day	WOMAC; VAS
Baek et al. [58]	Korea 2024	100	Ginger	Placebo	8 weeks	60.66 ± 6.87	60.54 ± 6.34	78/100	GGE03 1600 mg/day	WOMAC; VAS
Ardne NK et al. [59]	UK 2016	474	Vitamin D	Placebo	36 month	64.0 ± 8.0	64.0 ± 8.0	289/474	Cholecalciferol 800 IU/day	WOMAC
Jin XZ et al. [60]	Australia 2016	413	Vitamin D	Placebo	24 month	63.5 ± 6.9	62.9 ± 7.2	208/413	cholecalciferol 50,000 IU/day	WOMAC; VAS
McAlindon T et al. [19]	USA 2013	146	Vitamin D	Placebo	24 month	61.8 ± 7.7	63 ± 9.3	89/146	Cholecalciferol 2000 IU/day	WOMAC
Sanghi et al. [61]	India 2013	103	Vitamin D	Placebo	12 month	53.24 ± 9.64	53.00 ± 7.44	66/103	Cholecalciferol 60,000 IU/day	WOMAC

**Table 2:** Network meta-analysis of WOMAC pain.

A							
−2.47 (−11.18, 5.8)	B						
1.88 (−7.05, 10.97)	4.32 (−3.8, 12.86)	C					
−0.11 (−9.81, 9.45)	2.32 (−6.37, 11.46)	−2 (−11.4, 7.32)	D				
−8.56 (−16.27, −0.7)	−6.02 (−12.71, 0.97)	−10.34 (−17.89, −2.92)	−8.35 (−16.73, −0.11)	E			
−0.71 (−9.49, 8.21)	1.75 (−6.09, 10.04)	−2.54 (−11.19, 6.03)	−0.53 (−9.94, 8.77)	7.82 (0.51, 15.06)	F		
−0.7 (−10.24, 8.71)	1.84 (−6.89, 10.49)	−2.53 (−11.8, 6.47)	−0.5 (−10.77, 9.18)	7.78 (−0.28, 15.77)	0.02 (−9.15, 8.8)	G	
2.08 (−4.33, 8.71)	4.55 (−0.57, 10.15)	0.22 (−5.87, 6.44)	2.23 (−4.9, 9.32)	**10.58 (6.45, 14.78)**	2.78 (−3.15, 8.75)	2.77 (−3.93, 9.76)	H

Bold formatting to the data with statistical significance. A: Eggshell membrane; B: curcumin; C: collagen; D: krill oil; E: Boswellia; F: ginger; G: vitamin D; H: placebo.

**Table 3:** Network meta-analysis of WOMAC stiffness.

A							
0.32 (−5.45, 6.12)	B						
−0.07 (−6.12, 6.2)	−0.38 (−5.82, 5.21)	C					
−1.01 (−7.7, 5.81)	−1.31 (−7.53, 4.84)	−0.93 (−7.45, 5.54)	D				
−8.13 (−13.74, −2.64)	−8.46 (−13.21, −3.74)	−8.06 (−13.52, −2.97)	−7.11 (−13.21, −1.33)	E			
−0.77 (−6.85, 5.29)	−1.07 (−6.63, 4.45)	−0.7 (−6.65, 5.03)	0.25 (−6.43, 6.7)	7.37 (2.12, 12.65)	F		
−0.1 (−6.86, 6.98)	−0.36 (−6.73, 6.04)	0.03 (−6.63, 6.73)	0.94 (−6.26, 8.14)	8.07 (2.1, 14.31)	0.68 (−5.77, 7.47)	G	
1.32 (−3.13, 6.06)	1.02 (−2.54, 4.78)	1.4 (−2.78, 5.5)	2.33 (−2.66, 7.36)	**9.47 (6.39, 12.74)**	2.1 (−1.99, 6.41)	1.37 (−3.79, 6.69)	H

Bold formatting to the data with statistical significance. A: Eggshell membrane; B: curcumin; C: collagen; D: krill oil; E: Boswellia; F: ginger; G: vitamin D; H: placebo.

**Table 4:** Network meta-analysis of WOMAC function.

A							
−5.4 (−18.82, 7.65)	B						
−4.87 (−18.75, 8.88)	0.53 (−12.14, 13.66)	C					
−9.46 (−24.1, 5.1)	−4.12 (−17.58, 10)	−4.59 (−18.99, 9.77)	D				
−9.42 (−21.19, 2.53)	−4.08 (−14.46, 6.96)	−4.55 (−15.75, 7)	0.06 (−12.45, 12.66)	E			
−0.54 (−14.14, 13.39)	4.9 (−7.53, 17.5)	4.43 (−8.94, 17.59)	8.96 (−5.33, 23.24)	8.89 (−2.27, 20.15)	F		
0.34 (−14.42, 14.64)	5.78 (−8.12, 19.31)	5.25 (−9.34, 19.16)	9.85 (−5.81, 24.68)	9.74 (−2.84, 21.84)	0.86 (−13.35, 14.49)	G	
4.51 (−5.42, 14.59)	**9.96 (1.44, 18.79)**	**9.42 (0.02, 19)**	**14.01 (3.37, 24.93)**	**14 (7.74, 20.21)**	5.05 (−4.24, 14.45)	4.21 (−6.04, 15.16)	H

Bold formatting to the data with statistical significance. A: Eggshell membrane; B: curcumin; C: collagen; D: krill oil; E: Boswellia; F: ginger; G: vitamin D; H: placebo.

**Table 5:** Visual analog scale.

**A**							
−4.01 (−24.14, 16.25)	B						
−8.43 (−29.11, 12.55)	−4.37 (−20.82, 12.4)	C					
3.35 (−20.83, 27.84)	7.4 (−13.35, 28.44)	11.65 (−9.64, 33.05)	D				
−8.94 (−28.14, 10.08)	−4.92 (−19.24, 9.39)	−0.61 (−16.03, 14.94)	−12.21 (−32.3, 7.75)	E			
−3.57 (−23.43, 16.36)	0.43 (−14.72, 16.08)	4.76 (−11.53, 21.43)	−6.96 (−27.22, 13.61)	5.4 (−8.82, 19.57)	F		
2.86 (−26.46, 31.87)	6.84 (−20.02, 34.24)	11.13 (−15.88, 38.52)	−0.5 (−30.85, 29.73)	11.86 (−14.15, 37.82)	6.48 (−20.14, 33.17)	G	
8.26 (−8.33, 25.17)	**12.34 (1.59, 23.34)**	**16.65 (4.32, 29.09)**	4.96 (−12.72, 22.35)	**17.26 (8.06, 26.52)**	**11.89 (1.01, 22.49)**	5.41 (−18.87, 29.73)	H

Bold formatting to the data with statistical significance. A: Eggshell membrane; B: curcumin; C: collagen; D: krill oil; E: Boswellia; F: ginger; G: vitamin D; H: placebo.

**Table 6:** Specific adverse events.

Literature Source	Experimental Group Intervention	Adverse Reactions in the Control Group	Adverse Reactions in the Experimental Group
Hewlings et al., 2019 [27]	Eggshell Membrane	One case of headache and one case of poor sleep quality.	N
Park et al., 2024 [29]	Eggshell Membrane	One case of rash and itching occurring on the limbs and back.	N
Wang et al., 2020 [34]	Curcumin	One case each of nausea and vomiting, bloating, fatigue, drowsiness, sore throat with fever, and a sensation of fullness in the upper abdomen.	N
Wigler et al., 2003 [56]	Ginger	Two cases of heartburn	N
Jin XZ et al., 2016 [60]	Vitamin D	Four cases of hypercalcemia	Four cases of hypercalcemia

## Data Availability

The original contributions presented in this study are included in the article/Supplementary Material. Further inquiries can be directed to the corresponding author.

## Supplementary Materials

The following supporting information can be downloaded at https://www.mdpi.com/article/10.3390/nu17152547/s1: File S1: Equation search terms; File S2: Network meta-analysis code in R; File S3: SUCRA ranking in network meta-analysis; File S4: Egger’s test and trim-and-fill method in network meta-analysis; File S5: Risk of bias summary.
