# Supplementary material for: Comparative Effectiveness of Nutritional Supplements in the Treatment of Knee Osteoarthritis: A Network Meta-Analysis
Source: Nutrients. 2025 Aug 3;17(15):2547. doi: 10.3390/nu17152547 (PMC12348802; doi:10.3390/nu17152547)
Supplement: Supplementary file 1 [file nutrients-17-02547-s001.zip › Supplement.pdf]

Comparative Effectiveness of Nutritional Supplements in the Treatment of Knee Osteoarthritis: A Network Meta-Analysis

Supplementary File S1. Equation search terms

1) In Pubmed

(("Osteoarthritis, Knee"[Mesh]) OR (((((Osteoarthritis, Knee[Title/Abstract]) OR (Knee Osteoarthritides[Title/Abstract])) OR (Knee Osteoarthritis[Title/Abstract])) OR (Osteoarthritis of the Knee[Title/Abstract])) OR (Osteoarthritis of Knee[Title/Abstract]))) AND (("Curcumin"[Mesh]) OR (((((((((((Curcumin Phytosome[Title/Abstract]) OR (Phytosome, Curcumin[Title/Abstract])) OR (1,6-Heptadiene-3,5-dione, 1,7-bis(4-hydroxy-3-methoxyphenyl)-, (E,E)-[Title/Abstract])) OR (Diferuloylmethane[Title/Abstract])) OR (Turmeric Yellow[Title/Abstract])) OR (Yellow, Turmeric[Title/Abstract])) OR (Mervia[Title/Abstract])) OR (Zedoary[Title/Abstract])) OR (Curcuma domestica[Title/Abstract]))) OR (Curcuma longa[Title/Abstract])) OR (demethoxycurcumin[Title/Abstract])))

(("Osteoarthritis, Knee"[Mesh]) OR (((((Osteoarthritis, Knee[Title/Abstract]) OR (Knee Osteoarthritides[Title/Abstract])) OR (Knee Osteoarthritis[Title/Abstract])) OR (Osteoarthritis of the Knee[Title/Abstract])) OR (Osteoarthritis of Knee[Title/Abstract]))) AND ((("Vitamin D"[Mesh]) OR (Vitamin D[Title/Abstract])) OR (("Cholecalciferol"[Mesh]) OR (((((Cholecalciferol[Title/Abstract]) OR (Vitamin D 3[Title/Abstract])) OR ((3 beta,5Z,7E)-9,10-Secocholesta-5,7,10(19)-trien-3-ol[Title/Abstract])) OR (Calciol[Title/Abstract])) OR (Cholecalciferols[Title/Abstract]))))

(("Osteoarthritis, Knee"[Mesh]) OR (((((Osteoarthritis, Knee[Title/Abstract]) OR (Knee Osteoarthritides[Title/Abstract])) OR (Knee Osteoarthritis[Title/Abstract])) OR (Osteoarthritis of the Knee[Title/Abstract])) OR (Osteoarthritis of Knee[Title/Abstract]))) AND (("Euphausiacea"[Mesh]) OR ((((Euphausiacea[Title/Abstract]) OR (Nyctiphanes[Title/Abstract])) OR (Euphausia[Title/Abstract])) OR (Krill[Title/Abstract])))

(("Osteoarthritis, Knee"[Mesh]) OR (((((Osteoarthritis, Knee[Title/Abstract]) OR (Knee Osteoarthritides[Title/Abstract])) OR (Knee Osteoarthritis[Title/Abstract])) OR (Osteoarthritis of the Knee[Title/Abstract])) OR (Osteoarthritis of Knee[Title/Abstract]))) AND (("Egg Shell"[Mesh]) OR ((((((Egg Shell[Title/Abstract]) OR (Egg Shells[Title/Abstract])) OR (Shell, Egg[Title/Abstract])) OR (Shells, Egg[Title/Abstract])) OR (Eggshell[Title/Abstract])) OR (Eggshells[Title/Abstract])))

(("Osteoarthritis, Knee"[Mesh]) OR (((((Osteoarthritis, Knee[Title/Abstract]) OR (Knee Osteoarthritides[Title/Abstract])) OR (Knee Osteoarthritis[Title/Abstract])) OR (Osteoarthritis of the Knee[Title/Abstract])) OR (Osteoarthritis of Knee[Title/Abstract]))) AND (("Boswellia"[Mesh]) OR ((((((((boswellic acid[Title/Abstract]) OR (Boswellia serrata[Title/Abstract])) OR (Boswellia carteri[Title/Abstract])) OR (Boswellia carterii[Title/Abstract])) OR (Boswellia sacra[Title/Abstract])) OR (Shallaki[Title/Abstract])) OR (Aflapin[Title/Abstract])) OR (5-loxin[Title/Abstract])))

(("Osteoarthritis, Knee"[Mesh]) OR (((((Osteoarthritis, Knee[Title/Abstract]) OR (Knee Osteoarthritides[Title/Abstract])) OR (Knee Osteoarthritis[Title/Abstract])) OR (Osteoarthritis of the Knee[Title/Abstract])) OR (Osteoarthritis of Knee[Title/Abstract]))) AND (("Zingiber officinale"[Mesh]) OR (((Zingiber officinale[Title/Abstract]) OR (Ginger[Title/Abstract])) OR (Gingers[Title/Abstract])))

(("Osteoarthritis, Knee"[Mesh]) OR (((((Osteoarthritis, Knee[Title/Abstract]) OR (Knee Osteoarthritides[Title/Abstract])) OR (Knee Osteoarthritis[Title/Abstract])) OR (Osteoarthritis of the Knee[Title/Abstract])) OR (Osteoarthritis of Knee[Title/Abstract]))) AND (((((((((((((Collagen[Title/Abstract]) OR (Collagen Felt[Title/Abstract])) OR (Collagen Fleece[Title/Abstract])) OR (Collagenfleece[Title/Abstract])) OR (Microfibril Collagen Hemostat[Title/Abstract])) OR (Collagen Hemostat, Microfibril[Title/Abstract])) OR (Avicon[Title/Abstract])) OR (Avitene[Title/Abstract])) OR (Collastat[Title/Abstract])) OR (Dermodress[Title/Abstract])) OR (Pangen[Title/Abstract])) OR (alpha-Collagen[Title/Abstract])) OR (alpha Collagen[Title/Abstract])) OR (Zyderm[Title/Abstract]))

2）In Embase

Search number	Query
#1	'knee osteoarthritis'/exp
#2	'knee osteoarthritis':ab,ti OR 'arthrosis, knee':ab,ti OR 'femorotibial arthrosis':ab,ti OR gonarthrosis:ab,ti OR 'knee arthrosis':ab,ti OR 'knee joint arthrosis':ab,ti OR 'knee osteo-arthritis':ab,ti OR 'osteoarthritis, knee':ab,ti
#3	#1 OR #2
#4	'curcumin'/exp
#5	curcumin:ab,ti OR ('1, 7 bis':ab,ti AND '4 hydroxy 3 methoxyphenyl':ab,ti AND '1, 6 heptadiene 3, 5 dione':ab,ti) OR (bis:ab,ti AND '4 hydroxy 3 methoxycinnamoyl':ab,ti AND methane:ab,ti) OR curcumine:ab,ti OR nanocurc:ab,ti OR 'turmeric yellow':ab,ti OR diferuloylmethane:ab,ti
#6	#4 OR #5
#7	#3 AND #6
#8	'vitamin d'/exp
#9	'vitamin d':ab,ti

#10	#8 OR #9
#11	'colecalciferol'/exp
#12	cholecalciferol:ab,ti OR 'vitamin d 3':ab,ti OR ('3 beta,5z,7e':ab,ti AND '9,10 secocholesta 5,7,10':ab,ti AND 19:ab,ti AND 'trien 3 ol':ab,ti) OR calciol:ab,ti OR cholecalciferols:ab,ti
#13	#11 OR #12
#14	#10 OR #13
#15	#14 AND #3
#16	'krill oil'/exp
#17	'krill oil':ab,ti
#18	#16 OR #17
#19	#18 AND #3
#20	'egg shell'/exp
#21	'egg shell':ab,ti OR eggshell:ab,ti
#22	#20 OR #21
#23	#22 AND #3
#24	'boswellia'/exp
#25	'boswellia':ab,ti
#26	#24 OR #25
#27	#27 AND #3
#28	'ginger'/exp
#28	'ginger':ab,ti OR 'zingiber officinale':ab,ti OR 'zingiberis rhizoma':ab,ti OR 'zinziber officinale':ab,ti
#29	#29 OR #30
#30	#29 AND #3
#31	'collagen'/exp
#32	'collagen':ab,ti OR biocor:ab,ti OR collagel:ab,ti OR 'collagen horm':ab,ti OR collastypt:ab,ti OR collistat:ab,ti OR lyostypt:ab,ti OR medistat:ab,ti OR novacol:ab,ti OR phonogel:ab,ti
#33	#31 OR #32
#34	#33 AND #3

3) In the Cochrane Library

Search number	Query
#1	Osteoarthritis, Knee OR Knee Osteoarthritides; Knee Osteoarthritis; Osteoarthritis of Knee; Osteoarthritis of the Knee
#2	Curcumin ORMervia; Turmeric Yellow; Curcumin Phytosome; Phytosome, Curcumin; Diferuloylmethane; Yellow, Turmeric; 1,6-Heptadiene-3,5-dione, 1,7-bis(4-hydroxy-3-methoxyphenyl)-, (E,E)-
#3	Cholecalciferol OR Cholecalciferols; (3 beta,5Z,7E)-9,10-Secocholesta-5,7,10(19)-trien-3-ol; Vitamin D3; Calciol; Vitamin D 3 OR Vitamin D

#4	Euphausiacea OR Euphausia; Krill; Nyctiphanes
#5	Egg Shell OR Eggshells; Eggshell; Egg Shells; Shells, Egg; Shell, Egg
#6	Boswellia OR Boswellia carteri; Boswellia sacra; Boswellia carterii; Boswellia serrata Shallaki Aflapin 5-loxin
#7	Zingiber officinale OR Ginger; Gingers
#8	Collagen OR Avicon; Microfibril Collagen Hemostat; Collagen Hemostat, Microfibril; alpha Collagen; alpha-Collagen; Collagen Fleece; Collagenfleece; Collagen Felt; Dermodress; Pangen; Avitene; Collastat; Zyderm
#9	#1 AND (#2 OR #3 OR #4 OR #5 OR #6 OR #7 or #8)

Supplementary File S2. Network meta-analysis code in R

```
install.packages("gemtc")
install.packages("rjags")
library(gemtc)
library(rjags)
# 安装并加载 readxl 包（如果尚未安装）
if (!require(readxl)) install.packages("readxl")
library(readxl)
# 设置文件路径
file_path <- "D:/Rstudio/连续性变量.xlsx"
# 读取 Excel 文件
data <- read_excel(file_path)
# 查看数据前几行，确认是否读取成功
head(data)
data$mean <- -data$mean network <- mtc.network(data.ab = data)
model <- mtc.model(
  network,
  type = "consistency",   # 一致性模型
  n.chain = 4,            # 设置 4 条链
  likelihood = "normal", # 似然函数为正态分布
  link = "identity",      # 连接函数为身份链接
  linearModel = "random" # 随机效应模型）
results <- mtc.run(model, n.adapt = 5000, n.iter = 50000, thin = 10)

# sucra 排序
sucraranks <- sucra(ranks)
print(sucraranks)
plot(sucraranks)

# 排序排名柱状图（非蜂窝图）
plot(ranks, col=palette(),
      font.axis=2,
```

```
        beside=T)
#  排序结果（一致性模型）
ranks <- rank.probability(results, preferredDirection = 1)
print(ranks)
write.csv(ranks, "ranks.csv")   #  数据导出到 Excel
gelman.plot(results)  收敛图   plot(results)轨迹图和密度图  summary(results)   #  查看结果

#  原版森林图
forest(results)

install.packages("netmeta")library(netmeta)
colnames(data) <- c("study", "treat", "mean_val", "sd_val", "n_val")
pw_df <- data.frame(
  TE = unlist(pw$TE),
  seTE = unlist(pw$seTE),
  study = unlist(pw$studlab),
  treat1 = unlist(pw$treat1),
  treat2 = unlist(pw$treat2)
)
pw <- pairwise(treat = treat,
               mean = mean_val,
               sd = sd_val,
               n = n_val,
               studlab = study,
               data = data,
               sm = "MD")
base_model <- rma(yi = TE, sei = seTE, data = pw_df, method = "REML")
tf_model <- trimfill(base_model)
res <- rma(yi = TE, sei = seTE, data = pw_df, method = "REML")
taf <- trimfill(res)
funnel(taf, main = "Trim-and-Fill Funnel Plot")
funnel(taf,
       main = "Trim-and-Fill Funnel Plot",
       cex = 0.6)   #  缩小圆点大小
```

Supplementary File S3.SUCRA ranking in network meta-analysis

PAIN: A；0.4324500 B:0.6629714 C.0.2729429 D:0.4495357 E:0.9808429

F:0.5028929 G:0.4976643 H:0.2007000

STIFFNESS:A:0.4335500 B:0.3911143 C:0.4467000 D:0.5525071

E:0.9965571F :0.5373929 G:0.4393000 H:0.2028786

Function：A:0.34520000B: 0.62895000 C:0.59838571 D:0.80848571

E:0.84200714 F:0.36780000 G:0.32940714 H:0.07976429

Vas：A:0.4433714B: 0.6008143C: 0.7661857D: 0.3260857E: 0.8027857

F:0.5778357G: 0.3681929H: 0.1147286

A = Eggshell membrane

B= Curcumin

C = Collagen

D = Krill oil

E = Boswellia

F = Ginger

G = Vitamin D

H = Placebo

Supplementary File S4.Egger's test and trim-and-fill method in network meta-analysis

Womac pain

```
Regression Test for Funnel Plot Asymmetry

Model:     mixed-effects meta-regression model
Predictor: standard error

Test for Funnel Plot Asymmetry: z = -3.8791, p = 0.0001
```

```
Limit Estimate (as sei -> 0):   b = -1.8651 (CI: -4.2837, 0.5535)

Random-Effects Model (k = 35; tau^2 estimator: REML)

   logLik   deviance        AIC
-117.4768   234.9536   238.9536
      BIC       AICc
 242.0063   239.3407

tau^2 (estimated amount of total heterogeneity): 41.5293 (SE = 10.9957)
tau (square root of estimated tau^2 value):      6.4443
I^2 (total heterogeneity / total variability):   99.78%
H^2 (total variability / sampling variability):  461.63

Test for Heterogeneity:
Q(df = 34) = 1253.9195, p-val < .0001

Model Results:

estimate      se     zval    pval    ci.lb    ci.ub
 -4.8377  1.1520  -4.1995  <.0001  -7.0956  -2.5799  ***

---
Signif. codes:  0 ‘***’ 0.001 ‘**’ 0.01 ‘*’ 0.05 ‘.’ 0.1 ‘ ’ 1
```

Womac Stiffness

```
Regression Test for Funnel Plot Asymmetry

Model:     mixed-effects meta-regression model
Predictor: standard error

Test for Funnel Plot Asymmetry: z = -5.1728, p < .0001
Limit Estimate (as sei -> 0):   b = -0.9736 (CI: -2.5117, 0.5646)

Estimated number of missing studies on the right side: 5 (SE = 3.8671)

Random-Effects Model (k = 39; tau^2 estimator: REML)

   logLik   deviance        AIC
-131.1854   262.3708   266.3708
      BIC       AICc
 269.6459   266.7136

tau^2 (estimated amount of total heterogeneity): 46.1623 (SE = 11.8661)
tau (square root of estimated tau^2 value):      6.7943
```

```
I^2 (total heterogeneity / total variability):   99.90%
H^2 (total variability / sampling variability):  967.42

Test for Heterogeneity:
Q(df = 38) = 791.0303, p-val < .0001

Model Results:

estimate      se     zval    pval    ci.lb   ci.ub
 -2.2270  1.1634  -1.9142  0.0556  -4.5073  0.0532  .

---
Signif. codes:  0 ‘***’ 0.001 ‘**’ 0.01 ‘*’ 0.05 ‘.’ 0.1 ‘ ’ 1
```

Womac Function

```
Regression Test for Funnel Plot Asymmetry

Model:     mixed-effects meta-regression model
Predictor: standard error

Test for Funnel Plot Asymmetry: z = -3.0201, p = 0.0025
Limit Estimate (as sei -> 0):   b = -5.9534 (CI: -9.8500, -2.0567)

Estimated number of missing studies on the right side: 0 (SE = 3.5840)

Random-Effects Model (k = 35; tau^2 estimator: REML)

   logLik   deviance        AIC
-130.7796   261.5591   265.5591
      BIC       AICc
 268.6119   265.9462

tau^2 (estimated amount of total heterogeneity): 77.6634 (SE = 21.2219)
tau (square root of estimated tau^2 value):      8.8127
I^2 (total heterogeneity / total variability):   99.51%
H^2 (total variability / sampling variability):  204.14

Test for Heterogeneity:
Q(df = 34) = 2453.5400, p-val < .0001

Model Results:

estimate      se     zval    pval     ci.lb    ci.ub
```

```
 -9.5743  1.6149  -5.9287  <.0001  -12.7394  -6.4091  ***

---
Signif. codes:  0 ‘***’ 0.001 ‘**’ 0.01 ‘*’ 0.05 ‘.’ 0.1 ‘ ’ 1
```

Vas

```
Regression Test for Funnel Plot Asymmetry

Model:     mixed-effects meta-regression model
Predictor: standard error
Test for Funnel Plot Asymmetry: z = -2.4295, p = 0.0151
Limit Estimate (as sei -> 0):   b = -6.0723 (CI: -12.7910, 0.6464)

Estimated number of missing studies on the right side: 0 (SE = 3.0939)
Random-Effects Model (k = 26; tau^2 estimator: REML)
 logLik  deviance       AIC       BIC
-94.6950  189.3901  193.3901  195.8278
    AICc
193.9355

tau^2 (estimated amount of total heterogeneity): 105.0753 (SE = 31.6735)
tau (square root of estimated tau^2 value):      10.2506
I^2 (total heterogeneity / total variability):   99.98%
H^2 (total variability / sampling variability):  4556.06

Test for Heterogeneity:
Q(df = 25) = 3161.4578, p-val < .0001
Model Results:
estimate      se     zval    pval     ci.lb    ci.ub
-13.0327  2.0773  -6.2738  <.0001  -17.1042  -8.9612  ***

---Signif. codes:  0 ‘***’ 0.001 ‘**’ 0.01 ‘*’ 0.05 ‘.’ 0.1 ‘ ’ 1
```

Supplementary File S5. Risk of bias summary

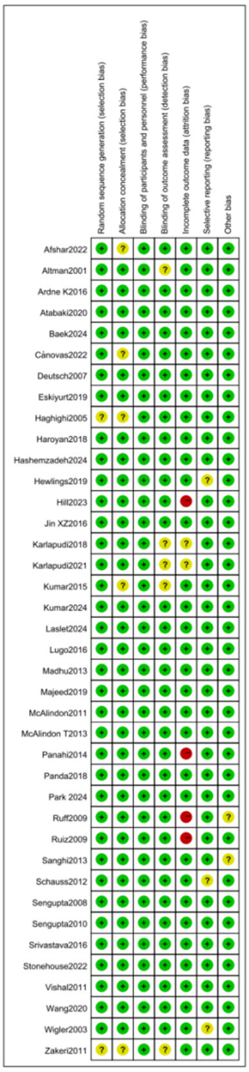
Random sequence generation (selection bias)
Allocation concealment (selection bias)
Blinding of participants and personnel (performance bias)
Blinding of outcome assessment (detection bias)
Incomplete outcome data (attrition bias)
Selective reporting (reporting bias)
Other bias
Afshar2022
Altman2001
Ardne K2016
Atabaki2020
Baek2024
Cánovas2022
Deutsch2007
Eskiyurt2019
Haghighi2005
Haroyan2018
Hashemzadeh2024
Hewlings2019
Hill2023
Jin XZ2016
Karlapudi2018
Karlapudi2021
Kumar2015
Kumar2024
Laslet2024
Lugo2016
Madhu2013
Majeed2019
McAlindon2011
McAlindon T2013
Panahi2014
Panda2018
Park 2024
Ruff2009
Ruiz2009
Sanghi2013
Schauss2012
Sengupta2008
Sengupta2010
Srivastava2016
Stonehouse2022
Vishal2011
Wang2020
Wigler2003
Zakeri2011

Figure S1. Preferred Reporting Items for Systematic reviews and Meta-Analysis (PRISMA) diagram.

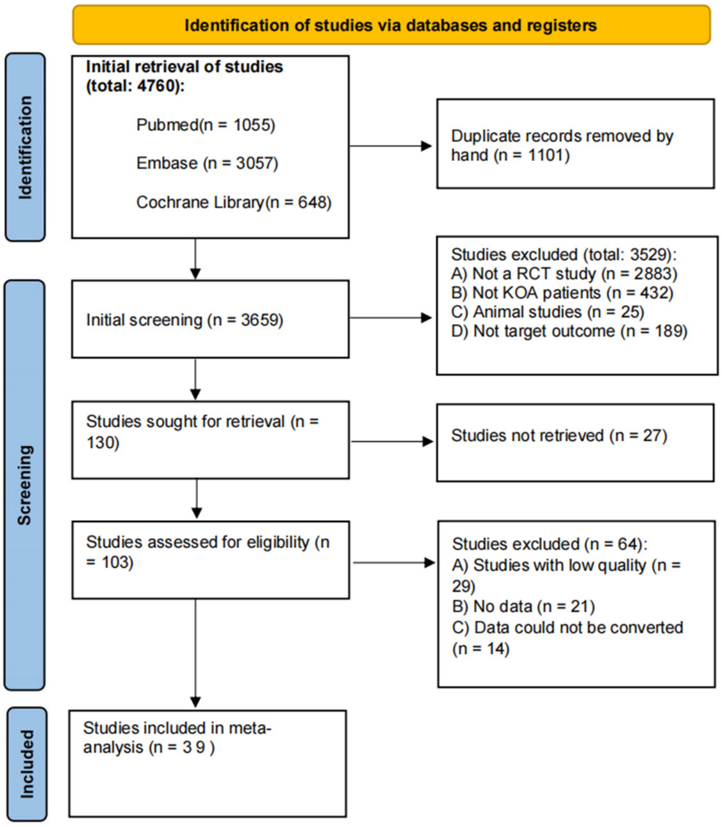

Figure S2. Risk of bias summary plot.

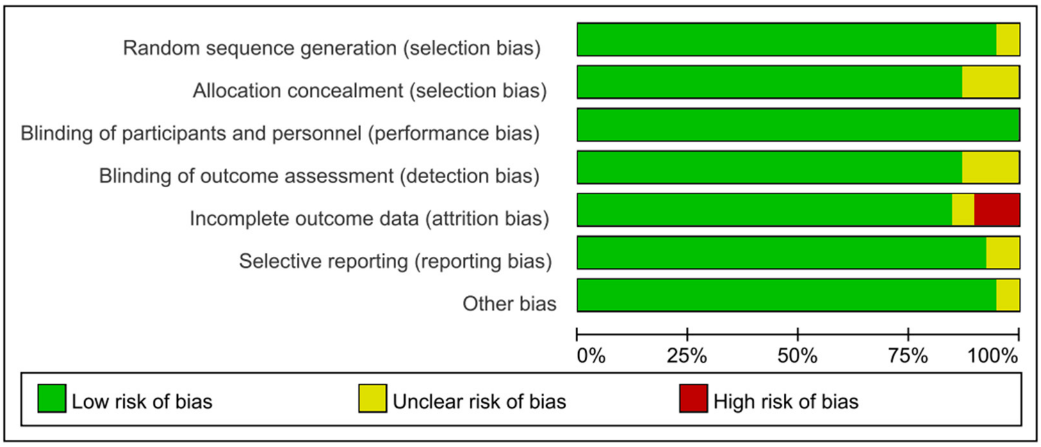

Figure S3. Network evidence plot.

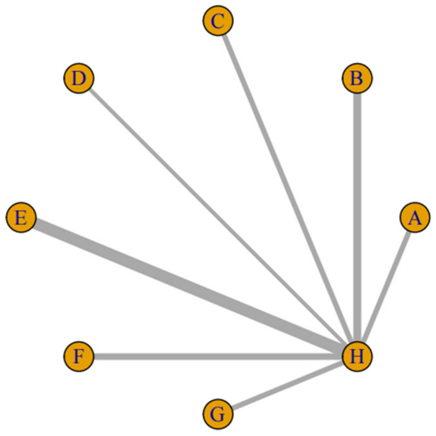

Womac pain

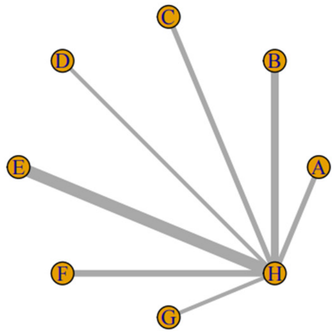

Womac stiffness

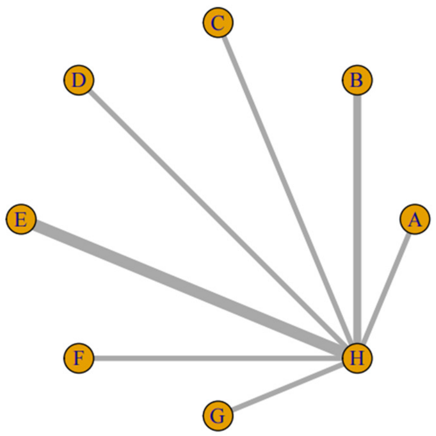

Womac function

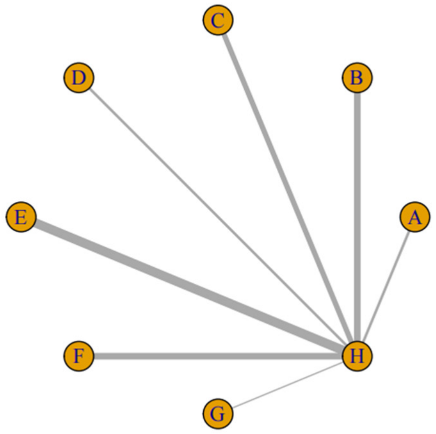

Vas

Figure S4. Probability ranking plots for each outcome measure.

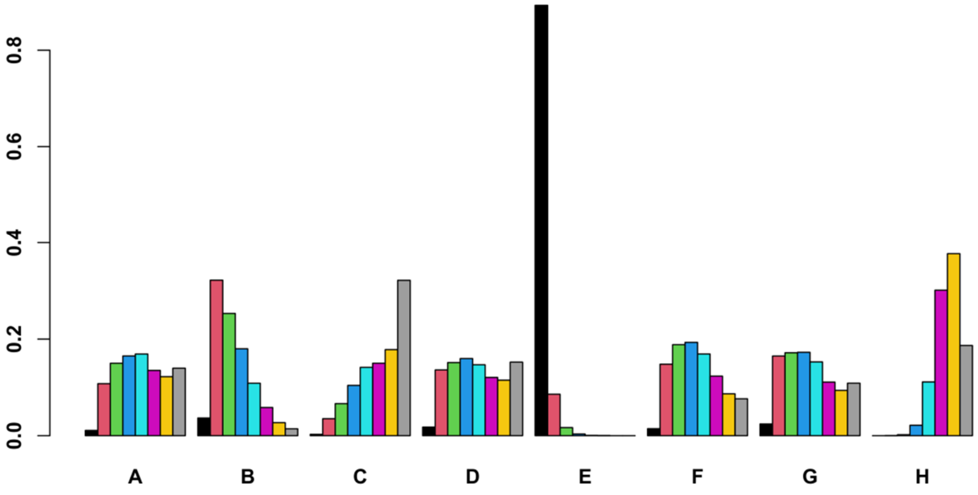

Womac pain

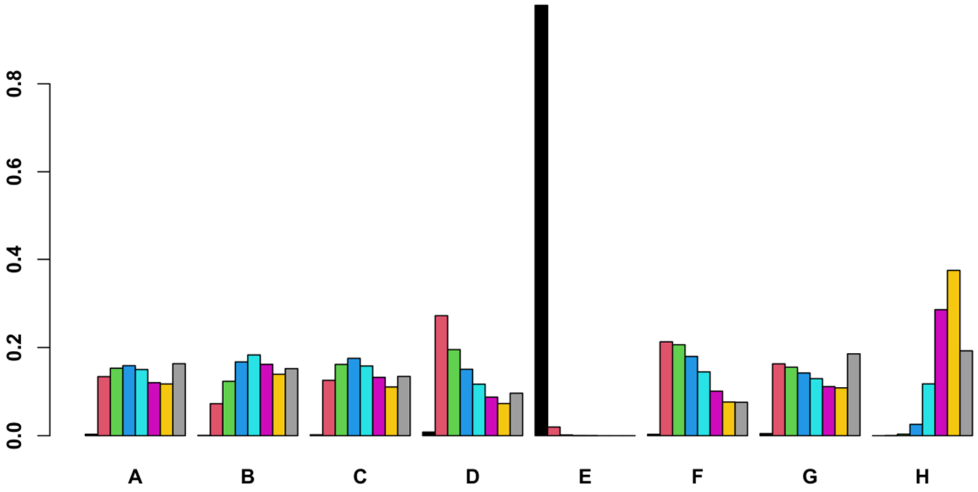

Womac stiffness

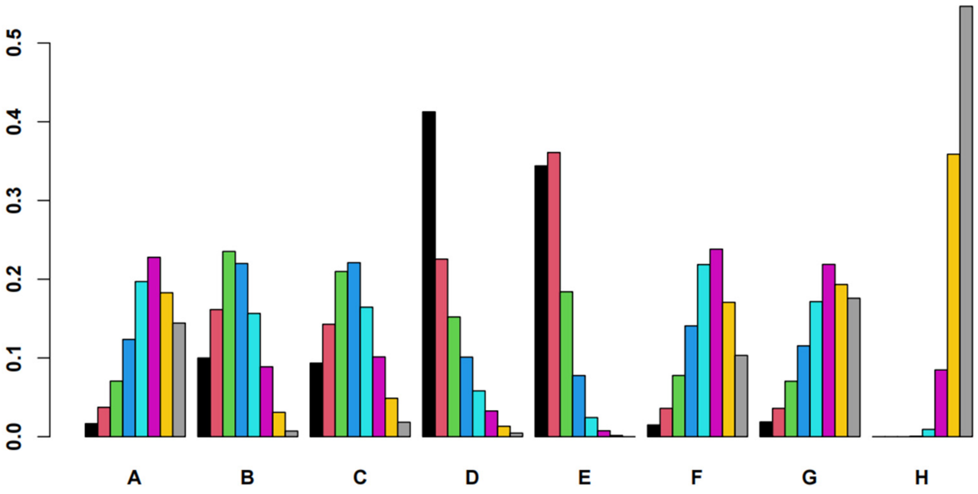

Womac function

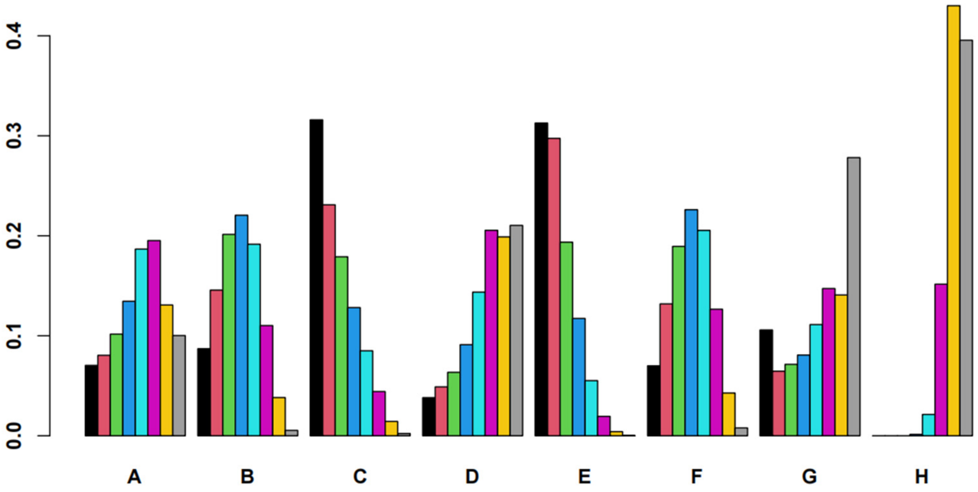

Vas

Figure S5. Adjusted funnel plot.

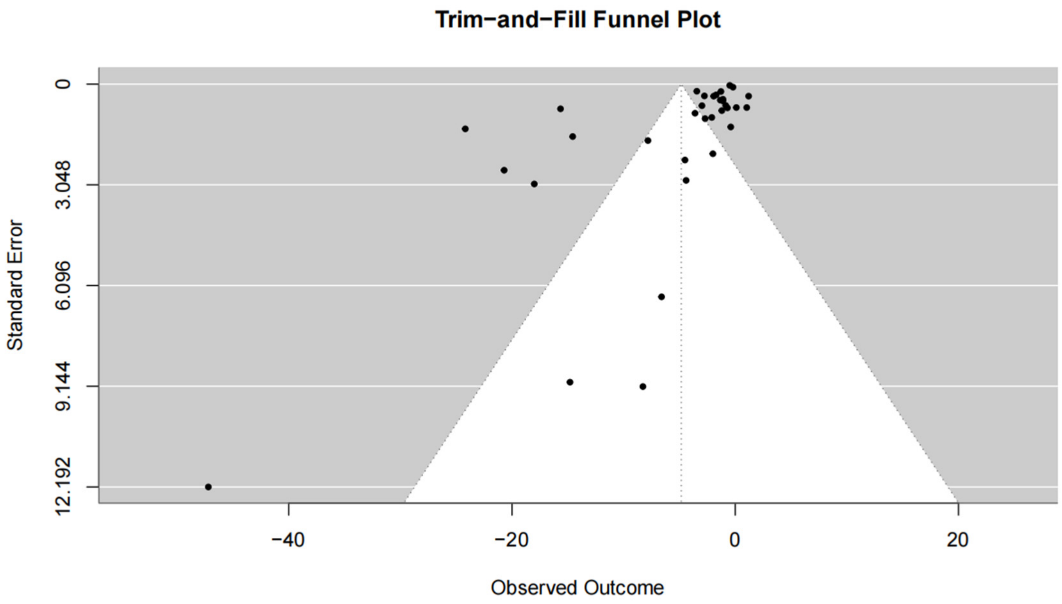

Womac pain

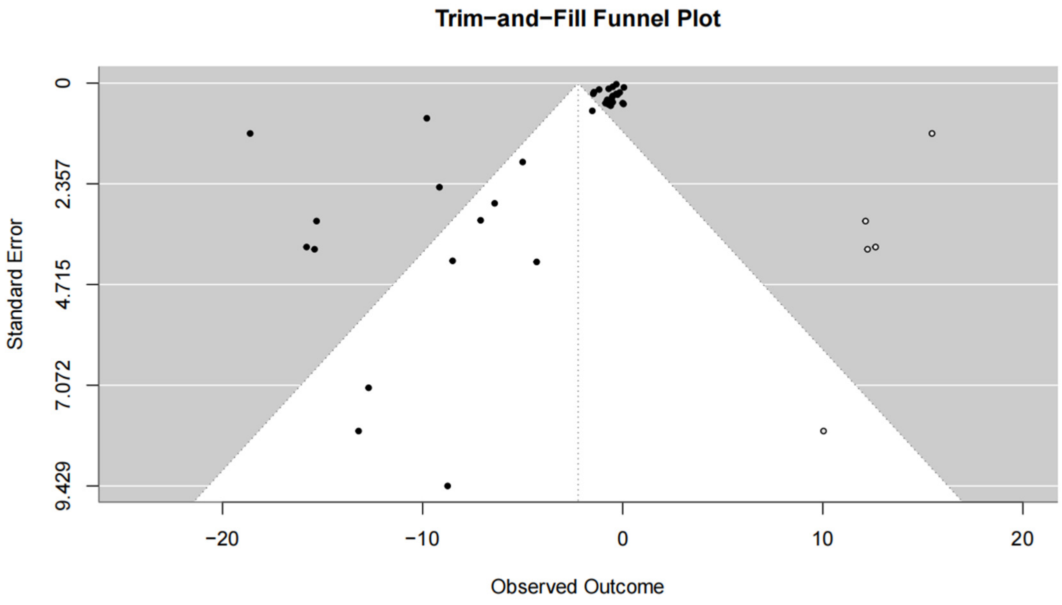

Womac stiffness

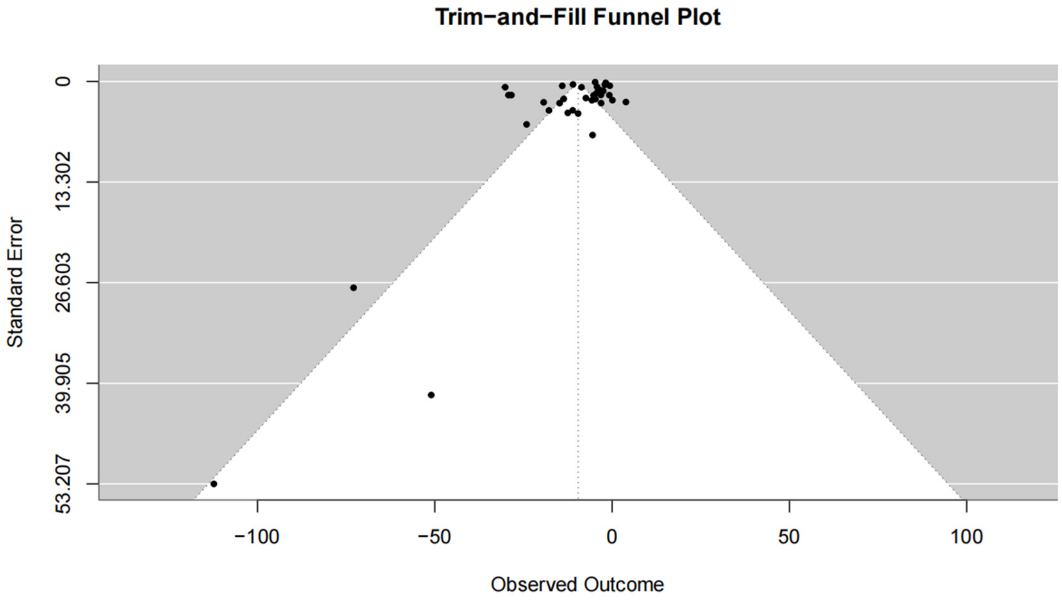

Womac function

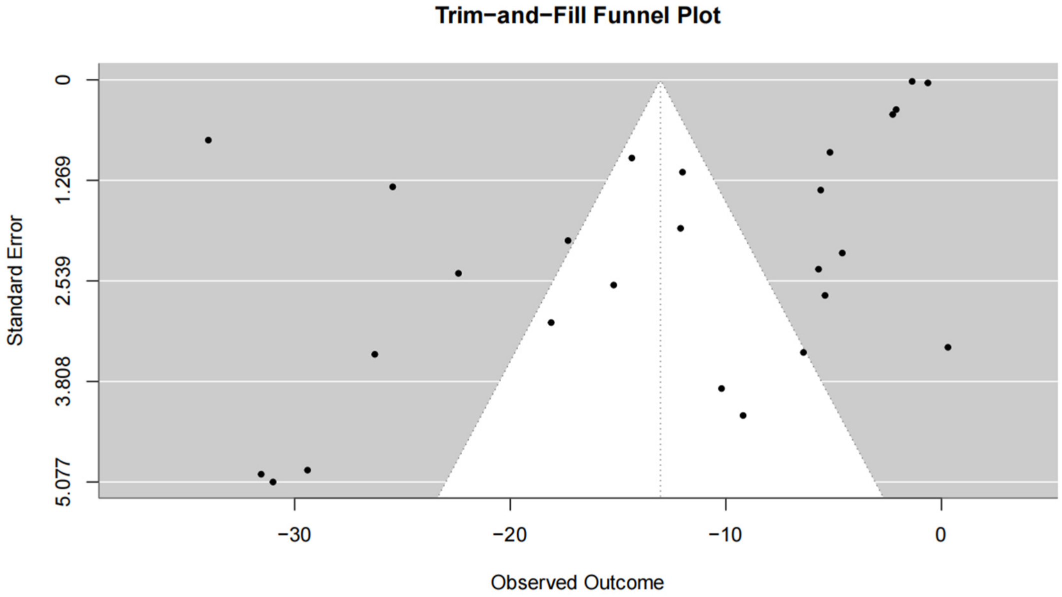

Vas

Table S1. Characteristics of the publications included in the meta-analysis.

Study	Country Year	N	Treatment	Control	Treatment duration	Age (years)		Female	Dosage	Outcome
						T	C			
Ruff et al[25]	USA2009	60	Eggshell	Placebo	60days	NR	NR	NR	NEM®500 mg/day	WOMAC
Cánovas et al[26]	Spain2022	51	Eggshell	Placebo	8 weeks	36.36±13.54	41.31±14.36	27/51	ESM®500 mg/day	VAS
Hewlings et al[27]	USA2019	88	Eggshell	Placebo	12weeks	NR	NR	63/88	BiovaFlex®450 mg/day	WOMAC
Eskiyurt et al[28]	Turkey2019	166	Eggshell	Placebo	90days	55.9±11.9	58.5±9.7	134/166	NEM®500 mg/day	WOMAC
Park et al[29]	Korea2024	99	Eggshell	Placebo	12weeks	57.73±7.75	58.54±8.28	79/99	NEM®500 mg/day	WOMAC:VAS
Hashemzadeh et al [30]	Iran2024	71	Curcumin	Placebo	6weeks	54.11±5.80	56.54±5.77	60/71	SinaCurcuminTM 80 mg/day	WOMAC
Madhu et al[31]	India2013	60	Curcumin	Placebo	6weeks	56.63±10.58	56.77±9.98	34/60	TurmacinTM 1000 mg/day	VAS
Panahi et al[32]	Iran2014	40	Curcumin	Placebo	6weeks	57.32±8.78	57.57±9.05	31/40	C3 Complex®1500 mg/day	WOMAC:VAS
Srivastava et al[33]	India2016	160	Curcumin	Placebo	16weeks	50.23±8.08	50.27±8.63	103/160	Haridra®1000 mg/day	WOMAC:VAS
Wang et al[34]	Australia2020	70	Curcumin	Placebo	12weeks	61.3±8.5	62.4±8.8	39/70	TurmacinTM 1000 mg/day	WOMAC:VAS
Atabaki et al[35]	Iran2020	30	Curcumin	Placebo	12weeks	49.13±8.87	48.26±7.81	30/30	SinaCurcumin®80 mg/day	VAS
Haroyan et al[36]	Armenia2018	134	Curcumin	Placebo	12weeks	54.65±8.84	56.04±8.55	127/134	CuraMed®1500 mg	WOMAC
Panda et al[37]	India2018	50	Curcumin	Placebo	12weeks	55.20±8.58	53.12±8.25	NR	Curene®500 mg	WOMAC:VAS
Benito-Ruiz et al[38]	Spain2009	207	Collagen	Placebo	24weeks	58.7±10.4	59.1±11.6	192/207	Colnatur®10g/day	WOMAC:VAS
Kumar et al[39]	India2014	60	Collagen	Placebo	13weeks	NR	NR	BCP:18/30<br>PCP:27/30	Pork Collagen Peptide 10g/day<br>Bovine Collagen Peptide 10g/day	VAS
Lugo et al[40]	USA2016	121	Collagen	Placebo	24weeks	53.5±7.9	53.1±7.8	60/121	UC-I®40mg/day	WOMAC:VAS
McAllindon et al[41]	USA2011	30	Collagen	Placebo	48weeks	58.9±8.0	60.3±8.5	18/30	Fortigel®10g/day	WOMAC
Schauss et al[42]	USA2012	88	Collagen	Placebo	10weeks	54.3±8.7	54.5±9.8	41/88	BioCell Collagen®2000mg/day	WOMAC
Stonehouse et al[43]	Australia2022	235	Krill il	Placebo	24weeks	59.9±6.3	59.3±6.6	77/235	Superba BoostT 4g/day	WOMAC
Laslet et al[44]	Australian2024	262	Krill il	Placebo	24weeks	61.7+9.3	61.4±9.9	122/262	krilloil softgel 2 g/day	WOMAC:VAS
Hill et al[45]	Korean2023	75	Krill il	Placebo	12weeks	57.0±10.28	59.0±11.82	44/75	FlexPro MD®600mg/day	WOMAC:VAS
Deutsch et al[46]	Canada2007	90	Krill il	Placebo	30days	54.6±14.8	55.3±14.3	43/90	NKOTM 300mg/day	WOMAC
Karlapudi et al[47]	India2018	70	Boswellia	Placebo	90days	48.7±1.13	50.3±1.34	43/70	L173014F2400 mg/day	WOMAC:VAS
Karlapudi et al[48]	India2021	67	Boswellia	Placebo	30days	51.60±8.48	51.81±7.21	50/67	Aflapin®100mg/day	WOMAC:VAS
Sengupta et al[49]	India2008	46	Boswellia	Placebo	90days	53.22±8.73	52.43±9.65	33/46	5-Loxin®250mg/day	WOMAC:VAS
Kumar et al[50]	India2024	80	Boswellia	Placebo	180days	48.60±7.39	47.93±7.89	47/80	Aflapin®100mg/day	WOMAC:VAS
Sengupta et al[51]	India2010	76	Boswellia	Placebo	90days	Aflapin®:53.2±7.9<br>5-Loxin®51.6±9.9	52.4±7.5	Aflapin®:22/38<br>5-Loxin®:26/38	5-Loxin®100 mg/day<br>Aflapin®100 mg/day	WOMAC:VAS
Vishal et al[52]	India2011	59	Boswellia	Placebo	30days	53.2±6.5	55.3±8.8	37/59	Aflapin®100mg/day	WOMAC<br>VAS
Haroyan et al[36]	Armenia2018	135	Boswellia	Placebo	12weeks	57.91±9.02	56.04±8.55	127/135	Curamin®1500mg/day	WOMAC
Majeed et al[53]	India2019	48	Boswellia	Placebo	120days	NR	NR	31/48	Boswellin®338.66 mg/day	WOMAC:VAS
Haghighi et al[54]	Iran2005	80	Ginger	Placebo	4weeks	58.3±0.33	58.4±0.36	23/80	Zingiber officinale 30mg/day	VAS
Zakeri et al[55]	Iran2011	204	Ginger	Placebo	6weeks	48.4±11.1	45.74±12.5	164/204	Zintoma®500mg/day	WOMAC:VAS
Altman et al[56]	USA2001	247	Ginger	Placebo	6weeks	64.0±11.5	66.3±11.6	152/247	EV.EXT 77510mg/day	WOMAC:VAS
Wigler et al[57]	Israel2003	29	Ginger	Placebo	12weeks	64.7(47-85)	59.3(42-81)	23/29	Zintona EC®1000mg/day	WOMAC
Afshar et al[58]	Iran2022	43	Ginger	Placebo	12weeks	55.62±8.646	54.86±6.63	29/43	G-Rup®60m/day	WOMAC:VAS
Baek et al[59]	Korea2024	100	Ginger	Placebo	8weeks	60.66±6.87	60.54±6.34	78/100	GGE031600mg/day	WOMAC:VAS
Ardne NK et al[60]	UK2016	474	Vitamin D	Placebo	36month	64.0±8.0	64.0±8.0	289/474	Cholecalciferol 800IU/day	WOMAC
Jin XZ et al[61]	Australia2016	413	Vitamin D	Placebo	24month	63.5±6.9	62.9±7.2	208/413	cholecalciferol 50000IU/day	WOMAC:VAS
McAlindon T et al[62]	USA2013	146	Vitamin D	Placebo	24month	61.8±7.7	63±9.3	89/146	Cholecalciferol 2000IU/day	WOMAC
Sanghi et al[63]	India2013	103	Vitamin D	Placebo	12month	53.24±9.64	53.00±7.44	66/103	Cholecalciferol 60000IU/day	WOMAC

Study	Country Year	N	Treatment	Control	Treatment duration	Age（years）		Female	Dosage	Outcome
						T	C			
Ruff et al 25	USA2009	60	Eggshell	Placebo	60days	NR	NR	NR	NEM® 500 mg/day	WOMAC
Cánovas et al 26	Spain2022	51	Eggshell	Placebo	8 weeks	36.36 ± 13.54	41.31 ± 14.36	27/51	ESM® 500 mg/day	VAS
Hewlings et al 27	USA2019	88	Eggshell	Placebo	12weeks	NR	NR	63/88	BiovaFlex® 450 mg/day	WOMAC
Eskiyurt et al 28	Turkey2019	166	Eggshell	Placebo	90days	55.9 ± 11.9	58.5 ± 9.7	134/166	NEM® 500 mg/day	WOMAC
Park et al 29	Korea2024	99	Eggshell	Placebo	12weeks	57.73 ± 7.75	58.54 ± 8.28	79/99	NEM® 500 mg/day	WOMAC；VAS
Hashemzadeh et al 30	Iran2024	71	Curcumin	Placebo	6weeks	54.11±5.80	56.54±5.77	60/71	SinaCurcumin™ 80 mg/day	WOMAC
Madhu et al 31	India2013	60	Curcumin	Placebo	6weeks	56.63 ± 10.58	56.77 ± 9.98	34/60	Turmacin™ 1000 mg/day	VAS
Panahi et al 32	Iran2014	40	Curcumin	Placebo	6weeks	57.32 ± 8.78	57.57 ± 9.05	31/40	C3 Complex® 1500 mg/day	WOMAC；VAS
Srivastava et al 33	India2016	160	Curcumin	Placebo	16weeks	50.23 ± 8.08	50.27 ± 8.63	103/160	Haridra® 1000 mg/day	WOMAC；VAS
Wang et al 34	Australia2020	70	Curcumin	Placebo	12weeks	61.3±8.5	62.4±8.8	39/70	Turmacin™ 1000 mg/day	WOMAC；VAS
Atabaki et al 35	Iran2020	30	Curcumin	Placebo	12weeks	49.13 ± 8.87	48.26 ± 7.81	30/30	SinaCurcumin® 80 mg/day	VAS

Haroyan et al 36	Armenia2018	134	Curcumin	Placebo	12weeks	54.65 ± 8.84	56.04 ± 8.55	127/134	CuraMed® 1500 mg	WOMAC
Panda et al 37	India2018	50	Curcumin	Placebo	12weeks	55.20±8.58	53.12±8.25	NR	Curene® 500 mg	WOMAC；VAS
Benito-Ruiz et al 38	Spain2009	207	Collagen	Placebo	24weeks	58.7±10.4	59.1±11.6	192/207	Colnatur® 10g/day	WOMAC；VAS
Kumar et al 39	India2014	60	Collagen	Placebo	13weeks	NR	NR	BCP:18/30<br>PCP:27/30	Pork Collagen Peptide 10g/day<br>Bovine Collagen Peptide 10g/day	VAS
Lugo et al 40	USA2016	121	Collagen	Placebo	24weeks	53.5 ± 7.9	53.1 ± 7.8	60/121	UC-II® 40mg/day	WOMAC；VAS
McAllindon et al 41	USA2011	30	Collagen	Placebo	48weeks	58.9 ± 8.0	60.3 ± 8.5	18/30	Fortigel® 10g/day	WOMAC
Schauss et al 42	USA2012	88	Collagen	Placebo	10weeks	54.3 ± 8.7	54.5 ± 9.8	41/88	BioCell Collagen® 2000mg/day	WOMAC
Stonehouse et al 43	Australia2022	235	Krill oil	Placebo	24weeks	59.9 ± 6.3	59.3 ± 6.6	77/235	Superba Boost™ 4g/day	WOMAC
Laslet et al 44	Australian2024	262	Krill oil	Placebo	24weeks	61.7±9.3	61.4±9.9	122/262	krill oil softgel 2 g/day	WOMAC；VAS
Hill et al 45	Korean2023	75	Krill oil	Placebo	12weeks	57.0 ± 10.28	59.0 ± 11.82	44/75	FlexPro MD® 600mg/day	WOMAC；VAS
Deutsch et al 46	Canada2007	90	Krill oil	Placebo	30days	54.6 ± 14.8	55.3 ± 14.3	43/90	NKO™ 300mg/day	WOMAC
Karlapudi et al 47	India2018	70	Boswellia	Placebo	90days	48.7 ± 1.13	50.3 ± 1.34	43/70	LI73014F2 400 mg/day	WOMAC；VAS

Karlapudi et al 48	India2021	67	Boswellia	Placebo	30days	51.60±8.48	51.81±7.21	50/67	Aflapin® 100mg/day	WOMAC；VAS
Sengupta et al 49	India2008	46	Boswellia	Placebo	90days	53.22 ± 8.73	52.43 ± 9.65	33/46	5-Loxin® 250mg/day	WOMAC；VAS
Kumar et al 50	India2024	80	Boswellia	Placebo	180days	48.60±7.39	47.93±7.89	47/80	Aflapin® 100mg/day	WOMAC；VAS
Sengupta et al 51	India2010	76	Boswellia	Placebo	90days	Aflapin®;53.2 ± 7.9 5-Loxin® 51.6 ± 9.9	52.4 ± 7.5	Aflapin®；22/38 5-Loxin® ;26/38	5-Loxin® 100 mg/day Aflapin® 100 mg/day	WOMAC；VAS
Vishal et al 52	India2011	59	Boswellia	Placebo	30days	53.2 ± 6.5	55.3 ± 8.8	37/59	Aflapin® 100mg/day	WOMAC VAS
Haroyan et al 36	Armenia2018	135	Boswellia	Placebo	12weeks	57.91 ± 9.02	56.04 ± 8.55	127/135	Curamin® 1500mg/day	WOMAC
Majeed et al 53	India2019	48	Boswellia	Placebo	120days	NR	NR	31/48	Boswellin® 338.66 mg/day	WOMAC；VAS
Haghighi et al 54	Iran2005	80	Ginger	Placebo	4weeks	58.3 ± 0.33	58.4 ± 0.36	23/80	Zingiber officinale 30mg/day	VAS
Zakeri et al 55	Iran2011	204	Ginger	Placebo	6weeks	48.4 ± 11.1	45.74 ± 12.5	164/204	Zintoma® 500mg/day	WOMAC；VAS
Altman et al 56	USA2001	247	Ginger	Placebo	6weeks	64.0 6 11.5	66.3 6 11.6	152/247	EV.EXT 77 510mg/day	WOMAC；VAS
Wigler et al 57	Israel2003	29	Ginger	Placebo	12weeks	64.7 (47–85)	59.3 (42–81)	23/29	Zintona EC® 1000mg/day	WOMAC

Afshar et al 58	Iran2022	43	Ginger	Placebo	12weeks	55.62 ± 8.646	54.86 ± 6.63	29/43	G-Rup® 60ml/day	WOMAC；VAS
Baek et al 59	Korea2024	100	Ginger	Placebo	8weeks	60.66±6.87	60.54±6.34	78/100	GGE03 1600mg/day	WOMAC；VAS
Ardne NK et al 60	UK2016	474	Vitamin D	Placebo	36month	64.0±8.0	64.0±8.0	289/474	Cholecalciferol 800IU/day	WOMAC
Jin XZ et al 61	Australia2016	413	Vitamin D	Placebo	24month	63.5±6.9	62.9±7.2	208/413	cholecalciferol 50000IU/day	WOMAC；VAS
McAlindon T et al 62	USA2013	146	Vitamin D	Placebo	24month	61.8±7.7	63±9.3	89/146	Cholecalciferol 2000IU/day	WOMAC
Sanghi et al 63	India2013	103	Vitamin D	Placebo	12month	53.24 ± 9.64	53.00 ± 7.44	66/103	Cholecalciferol 60000IU/day	WOMAC

Table S2. Network meta-analysis of WOMAC pain.

A							
-2.47 (-11.18, 5.8)	B						
1.88 (-7.05, 10.97)	4.32 (-3.8, 12.86)	C					
-0.11 (-9.81, 9.45)	2.32 (-6.37, 11.46)	-2 (-11.4, 7.32)	D				
-8.56 (-16.27, -0.7)	-6.02 (-12.71, 0.97)	-10.34 (-17.89, -2.92)	-8.35 (-16.73, -0.11)	E			
-0.71 (-9.49, 8.21)	1.75 (-6.09, 10.04)	-2.54 (-11.19, 6.03)	-0.53 (-9.94, 8.77)	7.82 (0.51, 15.06)	F		
-0.7 (-10.24, 8.71)	1.84 (-6.89, 10.49)	-2.53 (-11.8, 6.47)	-0.5 (-10.77, 9.18)	7.78 (-0.28, 15.77)	0.02 (-9.15, 8.8)	G	
2.08 (-4.33, 8.71)	4.55 (-0.57, 10.15)	0.22 (-5.87, 6.44)	2.23 (-4.9, 9.32)	10.58 (6.45, 14.78)	2.78 (-3.15, 8.75)	2.77 (-3.93, 9.76)	H

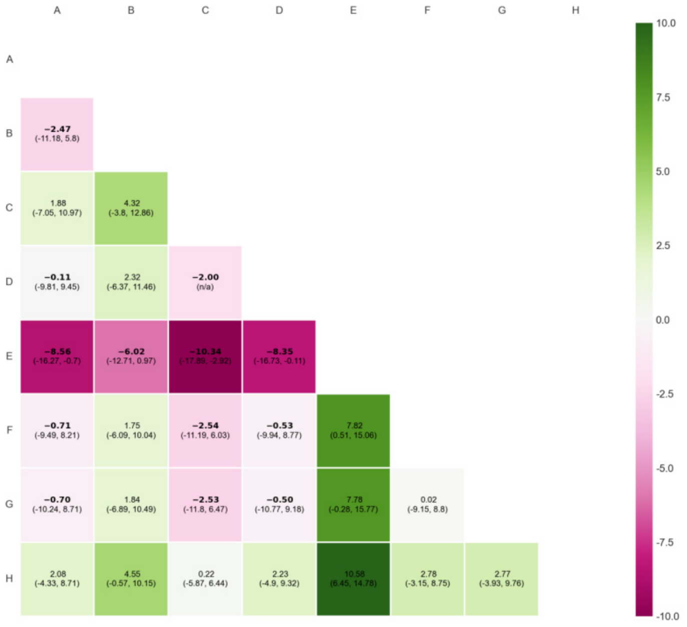

Table S3. Network meta-analysis of WOMAC stiffness.

A							
0.32 (-5.45, 6.12)	B						
-0.07 (-6.12, 6.2)	-0.38 (-5.82, 5.21)	C					
-1.01 (-7.7, 5.81)	-1.31 (-7.53, 4.84)	-0.93 (-7.45, 5.54)	D				
-8.13 (-13.74, -2.64)	-8.46 (-13.21, -3.74)	-8.06 (-13.52, -2.97)	-7.11 (-13.21, -1.33)	E			
-0.77 (-6.85, 5.29)	-1.07 (-6.63, 4.45)	-0.7 (-6.65, 5.03)	0.25 (-6.43, 6.7)	7.37 (2.12, 12.65)	F		
-0.1 (-6.86, 6.98)	-0.36 (-6.73, 6.04)	0.03 (-6.63, 6.73)	0.94 (-6.26, 8.14)	8.07 (2.1, 14.31)	0.68 (-5.77, 7.47)	G	
1.32 (-3.13, 6.06)	1.02 (-2.54, 4.78)	1.4 (-2.78, 5.5)	2.33 (-2.66, 7.36)	9.47 (6.39, 12.74)	2.1 (-1.99, 6.41)	1.37 (-3.79, 6.69)	H

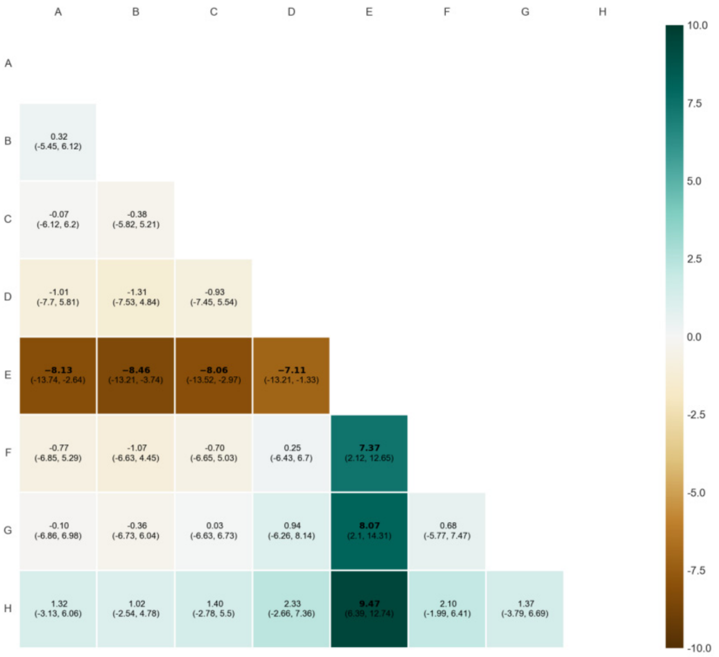

Table S4. Network meta-analysis of WOMAC function.

A							
−5.4 (−18.82, 7.65)	B						
−4.87 (−18.75, 8.88)	0.53 (−12.14, 13.66)	C					
−9.46 (−24.1, 5.1)	−4.12 (−17.58, 10)	−4.59 (−18.99, 9.77)	D				
−9.42 (−21.19, 2.53)	−4.08 (−14.46, 6.96)	−4.55 (−15.75, 7)	0.06 (−12.45, 12.66)	E			
−0.54 (−14.14, 13.39)	4.9 (−7.53, 17.5)	4.43 (−8.94, 17.59)	8.96 (−5.33, 23.24)	8.89 (−2.27, 20.15)	F		
0.34 (−14.42, 14.64)	5.78 (−8.12, 19.31)	5.25 (−9.34, 19.16)	9.85 (−5.81, 24.68)	9.74 (−2.84, 21.84)	0.86 (−13.35, 14.49)	G	
4.51 (−5.42, 14.59)	9.96 (1.44, 18.79)	9.42 (0.02, 19)	14.01 (3.37, 24.93)	14 (7.74, 20.21)	5.05 (−4.24, 14.45)	4.21 (−6.04, 15.16)	H

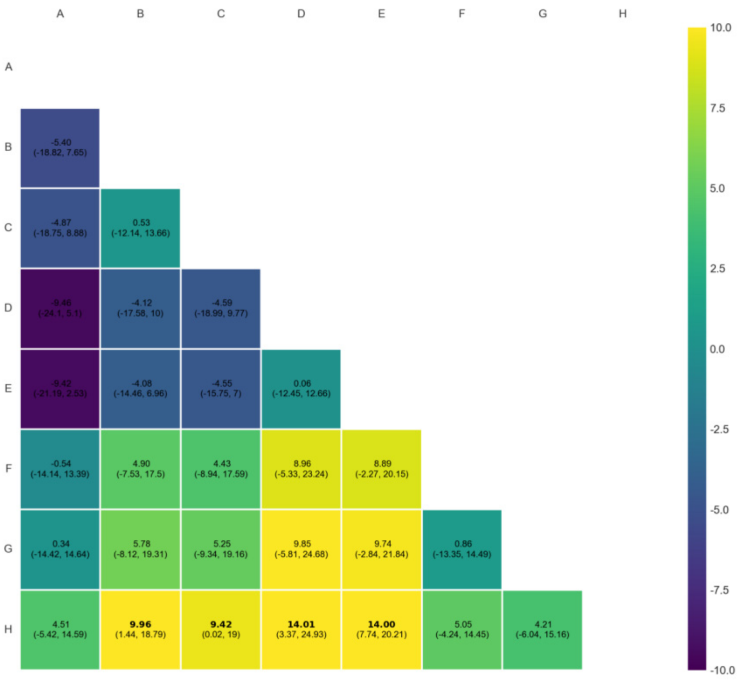

Table S5. Visual Analogue Scale.

A							
-4.01 (-24.14, 16.25)	B						
-8.43 (-29.11, 12.55)	-4.37 (-20.82, 12.4)	C					
3.35 (-20.83, 27.84)	7.4 (-13.35, 28.44)	11.65 (-9.64, 33.05)	D				
-8.94 (-28.14, 10.08)	-4.92 (-19.24, 9.39)	-0.61 (-16.03, 14.94)	-12.21 (-32.3, 7.75)	E			
-3.57 (-23.43, 16.36)	0.43 (-14.72, 16.08)	4.76 (-11.53, 21.43)	-6.96 (-27.22, 13.61)	5.4 (-8.82, 19.57)	F		
2.86 (-26.46, 31.87)	6.84 (-20.02, 34.24)	11.13 (-15.88, 38.52)	-0.5 (-30.85, 29.73)	11.86 (-14.15, 37.82)	6.48 (-20.14, 33.17)	G	
8.26 (-8.33, 25.17)	12.34 (1.59, 23.34)	16.65 (4.32, 29.09)	4.96 (-12.72, 22.35)	17.26 (8.06, 26.52)	11.89 (1.01, 22.49)	5.41 (-18.87, 29.73)	H

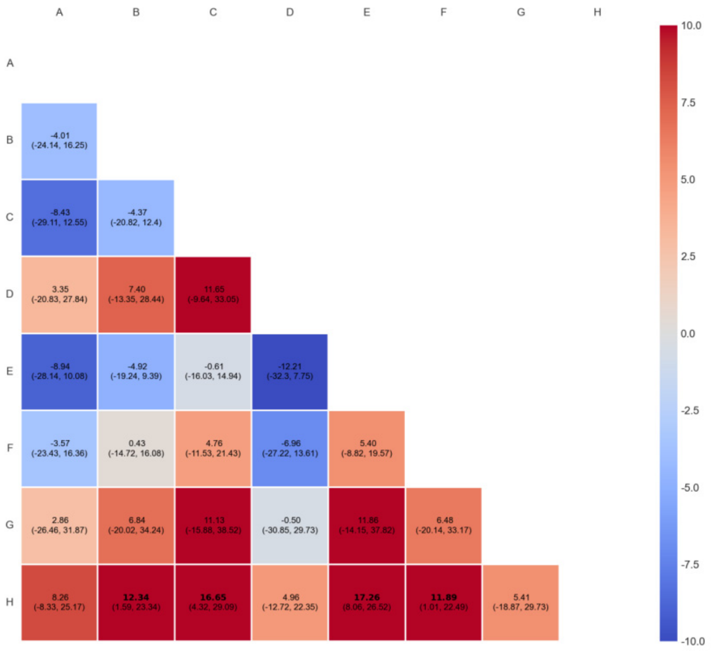

Table S6. Specific adverse events.

Literature source	Experimental group intervention	Adverse reactions in the control group	Adverse reactions in the experimental group
Hewlings et al 2019	Eggshell Membrane	One case of headache and one case of poor sleep quality.	N
Park et al 2024	Eggshell Membrane	One case of rash and itching occurring on the limbs and back.	N
Wang et al 2020	Curcumin	One case each of nausea and vomiting, bloating, fatigue, drowsiness, sore throat with fever, and a sensation of fullness in the upper abdomen.	N
Wigler et al 2003	Ginger	Two cases of heartburn	N
Jin XZ et al 2016	Vitamin D	Four cases of hypercalcemia	Four cases of hypercalcemia
